# Apospory appears to accelerate onset of meiosis and sexual embryo sac formation in sorghum ovules

**DOI:** 10.1186/1471-2229-11-9

**Published:** 2011-01-11

**Authors:** John G Carman, Michelle Jamison, Estella Elliott, Krishna K Dwivedi, Tamara N Naumova

**Affiliations:** 1 Plants, Soils & Climate Department, Utah State University, Logan, Utah 84322-4820, USA; 2 Caisson Laboratories, Inc., North Logan, Utah 84322-4820, USA; 3 College of Southern Idaho, Shields Building, P.O. Box 1238, Twin Falls, Idaho 83303, USA; 4 Nalichnaja Street 14 ap. 59, 199406 St. Petersburg, Russia

## Abstract

**Background:**

Genetically unreduced (2*n*) embryo sacs (ES) form in ovules of gametophytic apomicts, the 2*n *eggs of which develop into embryos parthenogenetically. In many apomicts, 2*n *ES form precociously during ovule development. Whether meiosis and sexual ES formation also occur precociously in facultative apomicts (capable of apomictic and sexual reproduction) has not been studied. We determined onset timing of meiosis and sexual ES formation for 569 *Sorghum bicolor *genotypes, many of which produced 2*n *ES facultatively.

**Results:**

Genotype differences for onset timing of meiosis and sexual ES formation, relative to ovule development, were highly significant. A major source of variation in timing of sexual germline development was presence or absence of apomictic ES, which formed from nucellar cells (apospory) in some genotypes. Genotypes that produced these aposporous ES underwent meiosis and sexual ES formation precociously. Aposporous ES formation was most prevalent in subsp. *verticilliflorum *and in breeding lines of subsp. *bicolor*. It was uncommon in land races.

**Conclusions:**

The present study adds meiosis and sexual ES formation to floral induction, apomictic ES formation, and parthenogenesis as processes observed to occur precociously in apomictic plants. The temporally diverse nature of these events suggests that an epigenetic memory of the plants' apomixis status exists throughout its life cycle, which triggers, during multiple life cycle phases, temporally distinct processes that accelerate reproduction.

## Background

For angiosperms, apomixis means asexual reproduction by seed [1]. It is strongly associated with hybridity and polyploidy, and molecular mechanisms responsible for it remain shrouded in complexity [2-4]. Apomixis involves the reprogramming of unreduced (2*n*) cells of the ovule, which thereafter follow a very different developmental trajectory than had the plant been sexual. Specifically, ovules of apomictic plants produce asexual totipotent cells. These form in the nucellus, chalaza or integuments, and embryos develop from them either directly (adventitious embryony) or after *2n *embryo sac (ES) formation (gametophytic apomixis). Apomictic (2*n*) ES usually resemble sexual ES, but embryony in them occurs parthenogenetically and often precociously. Whether in sexual plants or apomicts, embryony is the result of epigenome modifications that begin as early as floral transition [5,6].

Gametophytic apomixis is further divided into *i*) apospory, where the *2n *aposporous ES (AES) forms from a cell of the nucellus, chalaza or rarely an integument, and *ii*) diplospory, where the *2n *ES forms from an ameiotic megasporocyte (MMC). The formation of viable seed in apomicts requires the formation of functional endosperm, and this occurs pseudogamously or autonomously, i.e. with or without fertilization of the ES central cell, respectively. In adventitious embryony, a sexual ES with functional endosperm forms from which the developing adventitious embryo derives nutrients. The sexual embryo may survive and compete for nutrients with adventitious embryos [1,7].

Apomixis in angiosperms occurs in polyploids or polyhaploids and is found in 31 of 63 orders (compiled from [2] using APG III nomenclature [8]). Though widespread, it occurs infrequently, being reported in only 223 genera (of about 14,000), 41 of which belong to the Poaceae. Of these, 24 belong to the Panicoideae, which is a large and ancient subfamily of grasses many members of which, including *Sorghum *L. (but not *Zea *L.), have undergone few chromosome rearrangements and no whole genome duplications since a whole genome duplication occurred 65 million years ago that differentiated grasses from other monocots [9-11]. Accordingly, *Sorghum *is an anciently diploidized paleotetraploid (*n *= 10). It is divided into five subgenera, *Sorghum*, *Chaetosorghum*, *Heterosorghum*, *Parasorghum *and *Stiposorghum*. Subgenus *Sorghum *includes perennial *S. halapense *Pers. (2*n *= 4*× *= 40), perennial *S. propinquum *(Kunth) Hitchc. (2*n *= 2*× *= 20), and annual *S. bicolor *(L.) Moench (2*n *= 2*× *= 20). The latter is divided into subsp. *bicolor *(domesticated grain sorghums), subsp. *drummondii *(stabilized derivatives between grain sorghums and their closest wild relatives), and subsp. *verticilliflorum *(formerly subsp. *arundinaceum*, wild progenitors of grain sorghum). Subspecies *bicolor *is further divided into five races, bicolor, guinea, caudatum, kafir and durra, and 10 intermediate races [12].

Low frequency AES formation occurs in several subsp. *bicolor *lines [13-17]. However, none of the reports provide convincing molecular or cytological evidence of parthenogenesis, and claims to the contrary have met with skepticism [18,19]. In this respect, Gustafsson [20] reviewed evidence from several species that the *2n *egg in an AES from a plant that rarely produces AES may not be capable of parthenogenesis, an opinion shared by Asker and Jerling [21]. Nevertheless, the interrelatedness of Panicoideae [22] suggests that the AES formation observed in *S. bicolor *may be symplesiomorphic with that observed in the fully functional aposporous Panicoideae.

In practice sexual and apomictic plants are differentiated by *i*) cytological analyses of ovule development [23], *ii*) progeny tests using morphological or molecular markers [24], and *iii*) flow cytometry of seed nuclei to identify distinguishing embryo to endosperm ploidy level ratios [25]. However, several less-distinct traits also differentiate many apomicts from their related sexuals. For example in diplosporous species of *Tripsacum *L. [26,27] and *Elymus *L. [28], onset of *2n *ES formation, relative to stage of ovule development, occurs prior to onset of meiosis in related sexuals. Whether this is a general phenomenon of diplospory has not been investigated. In aposporous apomicts, the potentially competitive sexual germline is usually terminated by apoptosis from the MMC stage to early sexual ES formation. AES formation is detected cytologically as early as the MMC stage to as late as ES maturation. Timing of apospory is not rigid, and much within species and within plant variation occurs [1,20,21,29]. Likewise, parthenogenesis occurs prior to flower opening in many apomicts. This has been observed in *Alchemilla *L., *Aphanes *L., *Taraxacum *Cass., *Wikstroemia *Endl., *Ochna *L., *Allium *L., *Chondrilla *L., *Hieracium *L., *Crepis *L., *Potentilla *L., *Poa *L., *Elatostema *J. R. & G. Forst., *Tripsacum*, and *Parthenium *L. [20,21].

In the present study, we determined onset timing of megasporogenesis (female meiosis) and sexual ES formation relative to stage of ovule development for 569 genotypes from three populations of *S. bicolor*. We also determined the frequency of AES formation for each genotype. The genotypes were then grouped according to AES frequency, and the groups were compared based on onset timing of megasporogenesis and sexual ES formation. The results suggest that the apospory program in *S. bicolor *heterochronically accelerates, relative to stage of ovule development, the onset of meiosis and sexual ES formation.

## Results

### Ovary and ovule morphometrics

Regressions between ovary and ovule lengths at meiosis (dyad to early tetrad) and at the 1-nucleate ES (ES1) and early 8-nucleate ES (ES8) stages across 25 accessions were highly significant. However, the regression equations explained <50% of the variability (*r*^2^) at each stage (Additional file 1). Hence, large and small ovaries contained either large or small ovules, depending on accession, and ovary length only poorly predicted germline stage across accessions. For example, ovaries 0.3 cm long contained ovules in the meiocyte stage to the maturing ES stage depending on accession (Additional file 2).

**Figure 1:**
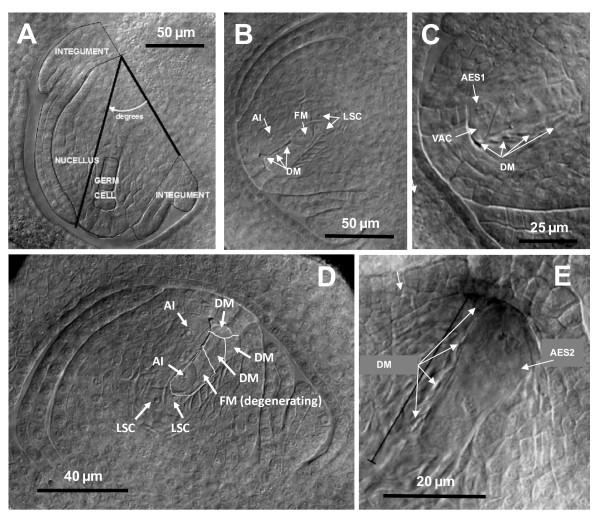
**Differential interference contrast images of cleared *Sorghum bicolor *ovules in sagittal section**. A) Procedure used to measure ovule area components (germ cell, nucellus and integuments), ovule curvature (angle), and inner integument length (distance from base to tip); from Carman [29], used with permission (caudatum, Agira, PI217855). B) Three degenerating megaspores (DM), the functional megaspore (FM), an aposporous initial (AI), and two large stack cells (LSC) (RIL, TX 37-6). C) Four DM and a vacuolate (VAC) 1-nucleate aposporous embryo sac (AES) (RIL, TX 152-6). D) Three DM, a degenerating FM, two AI, one of which is absorbing the FM, and two LSC (RIL, TX 4-7). E) Four DM and a 2-nucleate AES (breeding line, IS3620C).

Mean (±SE) ovule curvatures and areas (Figure 1A) were determined at two developmental stages, meiocyte and ES1, for 115 diploid genotypes and one naturally occurring tetraploid (Additional file 3). ANOVA was used to determine which of these two ovule development variables (curvature or area) would most closely correlate with germline stage (meiocyte or ES1). The dependent variable, coefficient of variation (CV), was represented by the CV values of 460 means, 115 for each of the four (2 × 2) method-by-stage combinations (diploid genotypes only). At the meiocyte and ES1 stages, mean CV values (±SE) based on ovule curvature were 0.151 (±0.004) and 0.134 (±0.004), respectively. The corresponding CV values based on ovule area were significantly larger, 0.210 (±0.006) and 0.185 (±0.005), respectively. The main effects (method and stage) were significant (*P *< 0.001), but the interaction effect was not significant. This analysis indicated that ovule curvature was less variable than ovule area at each germline stage.

**Figure 2:**
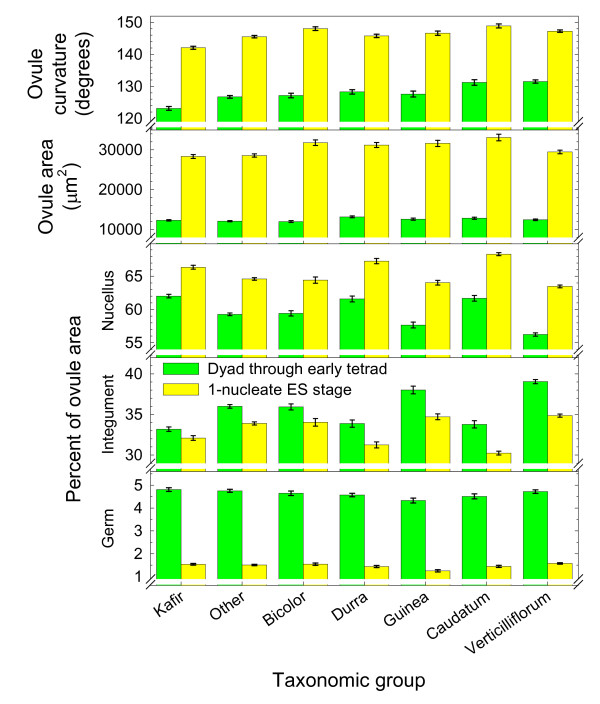
**Means (±SE) for ovule curvature, ovule area, and percentage of ovule area occupied by the nucellus, integument and germ (meiocyte or embryo sac) for seven taxonomic groups**. Measurements were taken at the meiocyte (dyad through early tetrad) and 1-nucleate embryo sac (ES) stages. See Additional file 3 for individual genotype data and Additional file 4 for ANOVA results.

Two sets of ANOVA were conducted to determine if variation in mean ovule curvature, ovule area, and three ovule area components (per genotype) varied according to taxonomic group. In the first set, all 116 genotypes from 57 accessions (Additional file 3) were partitioned into seven taxonomic groups, which consisted of the five subsp. *bicolor *races, accessions of subsp. *verticilliflorum*, and a group (other) that contained breeding lines and hybrids (Figure 2). Again, ovule curvature was more effective than ovule area in differentiating taxonomic groups, especially at the meiocyte stage. However, distinct partitioning also occurred among taxonomic groups based on the percentage of ovule area represented by the nucellus and integuments (Figure 2). These data further indicate that ovule shape (ovule curvature and relative growth dynamics of the nucellus and integuments) is more tightly correlated with germline development than is ovule area.

At the meiocyte stage, ovule curvature was most advanced for genotypes of the *verticilliflorum *group (Figure 2). In addition to strong curvature, the *verticilliflorum *group also had the largest and smallest percentages of ovule area represented by integuments and nucellus, respectively. As ovules mature, the integuments grow rapidly around the ovule, and consequently a larger proportion of the ovule is composed of integument. These data indicate that onset of meiosis was delayed in the *verticilliflorum *group compared to other groups (Figure 2). The opposite was observed for the kafirs. Here, ovules were only slightly curved at the onset of meiosis, and the integuments and nucellus represented the smallest and largest percentages of ovule area, respectively (Figure 2). Hence, in the kafirs, germline development is accelerated compared to other taxonomic groups. Variation within taxonomic group was also observed as indicated by highly significant (*P *< 0.001) effects for genotypes nested within taxonomic group and for genotypes nested within accessions (Additional file 4). The only insignificant effect was the taxonomic group by germline stage interaction for the percentage of ovule area represented by the germ (Figure 2, Additional file 4).

### Apospory in accessions and mapping populations

Nucellar cells normally die adjacent to the expanding embryo sac. In the present study, this progressive process of programmed nucellar cell death began shortly after megasporogenesis and continued until after fertilization when the nucellus was essentially consumed. In ovules of highly aposporous angiosperms, one or more nucellar cell(s) is re-programmed to undergo embryo sac formation. Early indications of this reprogramming include an abnormal doubling in size of the nucellar cell and nuclear enlargement [1,21]. In the present study, cells assuming these traits were counted as *i*) aposporous initials (AI) when they occurred in the micropylar region of the nucellus (usually adjacent to the MMC, meiocyte, or degenerating megaspores (DM)), or *ii*) large stack cells (LSC) when they occurred in the chalaza proximal to the MMC, meiocyte, or functional megaspore (FM) (Figure 1B, D). LSC developed from cells at the nucellus chalaza interface and belonged to or were closely associated with the cell file (stack) from which the MMC formed. Generally, LSC were much more prevalent than AI (Additional file 3).

We defined the FM stage as onset of FM enlargement, which coincided with DM degeneration (Figure 1B). We defined the 1-nucleate ES stage as acquisition by the FM of a vacuole similar in size to the nucleus. Likewise an AI was referred to as an AES once it had produced a similarly large vacuole. AES only rarely formed from LSC (based on observed locations of AES). Most were derived from AI and formed in the micropylar region. Sexual ES and AES were further characterized by number of nuclei present (Figure 1C, E).

Some AI, LSC and AES did not form until the FM stage. Hence, to minimize underestimating apospory, only ovules ranging in development from the FM stage through the ES2 stage were used in determining AI, AES and LSC frequencies. The ES2 stage criterion was used because determining the origin of the ES (sexual or aposporous) in ovules beyond the ES2 stage was problematic. In these ovules, megaspores and nucellar cells adjacent to the enlarging ES had degenerated.

**Figure 3:**
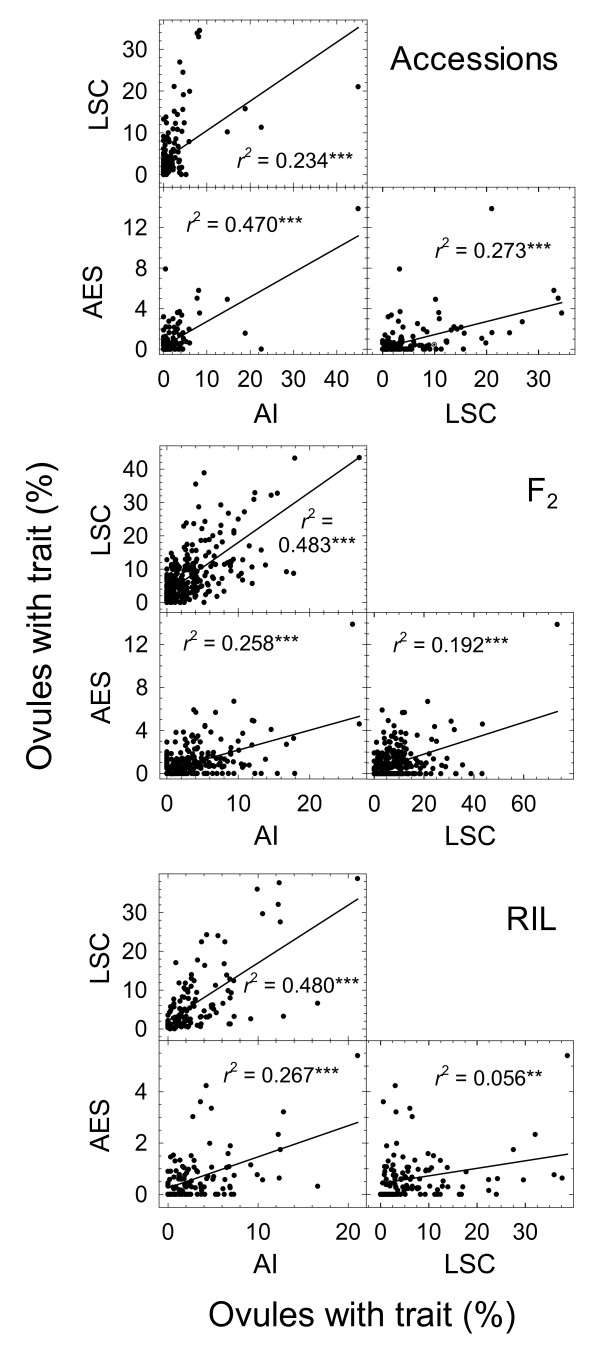
**Correlations between percentages of ovules containing large stack cells (LSC), aposporous embryo sacs (AES) and aposporous initials (AI)**. Points represent frequencies from 150 genotypes from 65 genetically diverse accessions, 300 genotypes from an F_2 _mapping population, and 119 genotypes from an F_8 _recombinant inbred line (RIL) population. For regression, ** and *** denote significance at *P *< 0.01 and *P *< 0.001, respectively.

Frequencies of AI, LSC and AES were determined for 150 *S. bicolor *genotypes from 65 accessions (Additional file 3, 116 genotypes; Additional file 5, 34 genotypes), a mapping population consisting of 300 F_2_, and a mapping population consisting of 119 recombinant inbred lines (RIL [30]). Correlations between AES and AI and between AES and LSC were higher among genotypes of the accessions than among genotypes of the mapping populations (Figure 3). In all three populations, the frequency of AES formation was more highly correlated with the frequency of AI formation than with the frequency of LSC formation. Compared to the genetically diverse accessions, regression *r*^2 ^values between LSC and AI were twice as high in the segregated F_2 _and RIL mapping populations (Figure 3). None of the regressions between percentage germline degeneration (measured for accessions only) and percentages of AI, AES or LSC (or combinations of these) was significant.

Eleven of the 150 diploid genotypes from 65 accessions exceeded 3% AES formation (Additional file 3). Five of these were from breeding lines of subsp. *bicolor *(5 of 30 lines) and five were from accessions of subsp. *verticilliflorum *(5 of 35 accessions). One, a caudatum, represented all other taxonomic groups (1 of 85 accessions). Two tests of equality of proportions were conducted. These matched the "other" group (1 of 85) against the breeding lines (5 of 30) and the "other" group against the *verticilliflorum *(5 of 35). Both tests were rejected (*P *< 0.001 and P < 0.01, respectively). Hence, apospory was most prevalent in wild land races of subsp. *verticilliflorum *and in breeding lines of subsp. *bicolor*.

**Figure 4:**
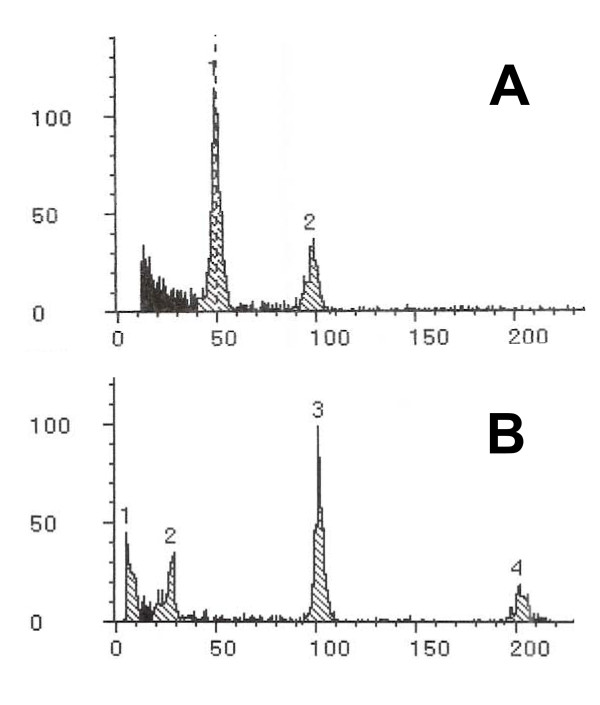
**Fluorescence intensity histograms of leaf tissue nuclei from diploid and tetraploid *Sorghum bicolor***. A) This histogram is from diploid subsp. *verticilliflorum*, accession IS11010, genotype 7.5d. B) This histogram is from a naturally occurring tetraploid plant from a typically diploid subsp. *verticilliflorum *accession, IS12702, genotype 76d.

Flow cytometry of leaf tissue was used to determine the ploidy of the 11 genotypes that exhibited ≥3% AES formation. Ten were diploid, but one, which exhibited the highest AES percentage (14% with 45% AI formation), was tetraploid (Figure 4). Three other genotypes of this accession (IS 12702, subsp. *verticilliflorum*) were diploid. These diploids had high AI levels relative to other accessions (Additional files 3, 5), but only one exhibited an AES frequency >3% (4.9%). Several other genotypes with >3% AES formation were from accessions in which multiple genotypes were analyzed but only one genotype exhibited the high AES level (Additional files 3, 5). Only two genotypes (from two different subsp. *verticilliflorum *accessions) exhibited >6% AES formation. Eight genotypes exhibited >6% AI formation, one caudatum, three from the breeding lines, and four from subsp. *verticilliflorum*.

### Apospory and ovule morphometrics

**Figure 5:**
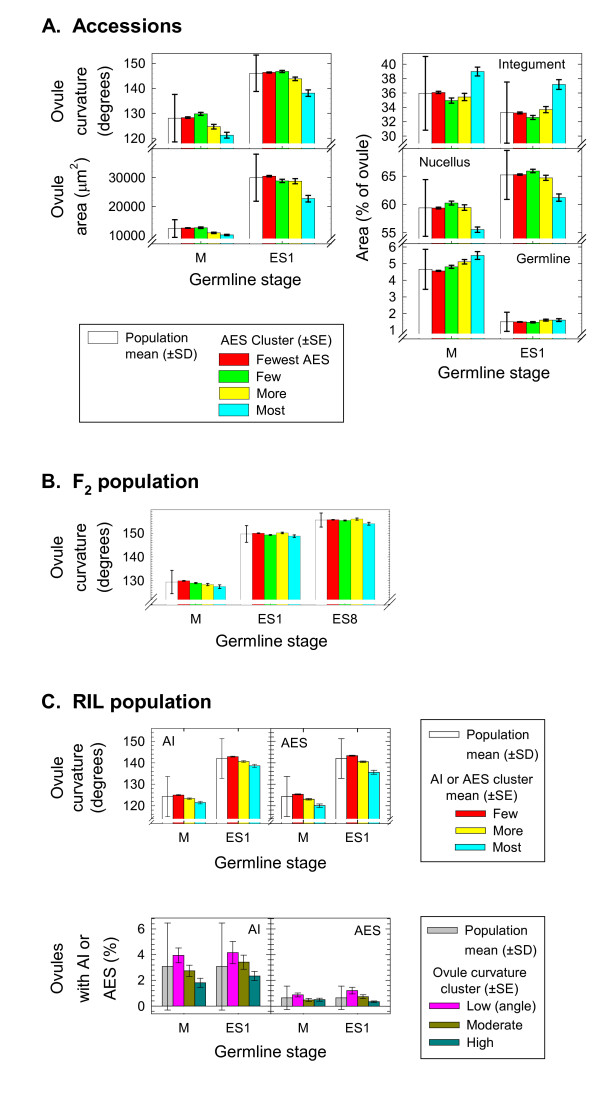
**Means for morphometric variables**. A) Mean ovule curvature, ovule area, and percentage ovule area occupied by integument, nucellus and germline (meiocyte or young embryo sac) for 115 diploid *S. bicolor *genotypes (population mean, ±SD) and for four groups of these genotypes partitioned by *k*-means clustering based on frequency of aposporous embryo sac (AES) formation (AES cluster, ±SE). Measurements were taken at the dyad through early tetrad (M) and the 1-nucleate embryo sac (ES1) stages. *k*-means clusters representing genotypes with the fewest, few, more and most AES consisted of 89, 15, 8 and 3 genotypes, respectively (see Additional file 6 for ANOVA results). B) Mean ovule curvature for 300 F_2 _*S. bicolor *genotypes (±SD) and for four groups of the F_2 _(±SE) partitioned by *k*-means clustering based on frequency AES formation. Measurements were taken at the M, ES1, and early 8-nucleate embryo sac (ES8) stages. *k*-means clusters representing F_2 _with the fewest, few, more and most AES consisted of 177, 89, 25 and 9 genotypes, respectively (see Additional file 7 for ANOVA results). C) Population (±SD) and cluster group (±SE) means based on 119 *S. bicolor *recombinant inbred lines (RIL). RIL were partitioned by *k*-means clustering based on frequency of AI or AES per genotype (top graphs). *k*-means clusters representing RIL with few, more and most AI or AES consisted of 81, 30 and 8 RIL or 76, 37 and 6 RIL, respectively. RIL were also partitioned by *k*-means clustering based on ovule curvature at the M and ES1 stages (bottom graphs). *k*-means clusters representing RIL with low, moderate and high ovule curvature angles at M or ES1 consisted of 49, 49 and 21 RIL or 19, 49 and 51 RIL, respectively (see Additional file 8 for ANOVA results).

An objective of the current study was to determine if tendencies for apospory in *S. bicolor *are associated with other morphometric ovule development variables. To accomplish this, *k-*means multivariate clustering was used to partition genotypes of accessions, F_2_, and RIL into 3-4 groups (per population) with similar frequencies of AI or AES. In all three populations, meiosis and sexual ES formation occurred precociously in the groups with the highest AES formation frequencies (Figure 5).

As noted above, 11 of 150 genotypes from 65 accessions exhibited an AES frequency >3%. Three of these grouped together to form the highest AES *k*-means cluster, and the remaining eight clustered together to form the second highest *k*-means group. Both groups underwent meiosis and sexual ES formation early (low ovule curvature values) compared to the other *k*-means groups (Figure 5A, see Additional file 6 for ANOVA results). Two of the three genotypes in the highest AES group were from a single breeding line and the third was a subsp. *verticilliflorum *genotype. In the second highest group (eight genotypes), three were from breeding lines, four were from subsp. *verticilliflorum *and one was a caudatum (subsp. *bicolor*). If earliness of meiosis and sexual ES formation promoted apospory, a higher frequency apospory should have been observed among the kafirs (Figure 2). However, the kafirs exhibited low AI and AES frequencies. In contrast, five of the 11 highest AES-forming genotypes belonged to subsp. *verticilliflorum*, which on average underwent meiosis later than most of the other taxonomic groups (Figure 2).

Ovule area values during meiosis were also significantly lower for the 11 highest frequency AES-forming genotypes (Figure 5A, more and most groups; Additional file 6). This was accompanied by significantly larger percentages of total ovule area represented by the meiocyte (Figure 5A, Germline). This indicates that in these relatively small non-curved ovules (of aposporously active genotypes), the sexual meiocyte was actively growing and dividing; and this occurred whether AES were present or not. In contrast, percentage values for ovule area represented by the nucellus and integuments for the two highest AES-forming groups were variable (Figure 5A). Note from Additional file 6 that variability among genotypes in clusters was significant. ANOVA were also performed for groups of genotypes defined by *k*-means clustering using AI frequencies, but significant differences in ovule curvature or area were not detected among these clusters.

Ovule curvature data for the meiocyte, ES1 and ES8 stages were collected for the 300 genotypes of the F_2 _mapping population (Figure 5B). As with the accessions, groups of F_2 _with the highest and the next to highest AES formation frequencies (nine and 25 genotypes, respectively) underwent meiosis earlier than the other groups. This precociousness persisted into the ES1 and ES8 stages only for genotypes from the highest AES formation group (Figure 5B, see Additional file 7 for ANOVA results). Mean ovule curvatures for *k*-means clusters based on AI frequencies did not differ significantly at any stage. Tests were conducted to determine if F_2 _plants with a low mean ovule curvature exhibited higher AES formation frequencies. For these tests, genotypes of the F_2 _population were clustered (*k*-means) by mean ovule curvature at the meiocyte, ES1 and ES8 stages, and ANOVA were performed to determine if differences existed among clusters in frequency of AES formation. The *F*-values for these analyses were not significant (Additional file 7).

Precociousness of meiosis and sexual ES formation in the highest AES and AI frequency clusters was more distinct among the well segregated F_8 _RIL (Figure 5C) than among the F_2 _(Figure 5B), and the degree of earliness in the two highest AES groups was similar to that observed among the genetically diverse accessions (Figure 5A). Genotypes with high AI frequencies generally had high AES frequencies (Figure 3). However, several exceptions were observed. Two of the eight RIL in the highest AI formation group were in the lowest AES formation group. Likewise one of six RIL in the high AES formation group was in the low AI formation group. Genotypes with several AI often did not exhibit AES formation, and some genotypes with relatively high AES formation apparently passed through the AI phase quickly as few AI were observed.

About 30% of the RIL clustered into the more and most AI and AES formation groups. In contrast, only about 10% of accessions and F_2 _clustered into the more and most groups. The high percentage of RIL in the high AES and AI formation groups affected the ovule curvature dynamics of the entire RIL population. This was detected by clustering RIL according to mean ovule curvature at the meiocyte and ES1 stages. Clusters of genotypes exhibiting the lowest ovule curvature values (developmentally precocious) exhibited significantly higher AI and AES frequencies (Figure 5C, see Additional file 8 for ANOVA results). As noted above, such analyses were not significant for the accessions or for the F_2 _population.

## Discussion

In grasses, a single ovule develops from the ovary placenta. Initially, the ovule primordium (young funiculus) grows inward and perpendicular to the inner ovary wall. As the ovule grows, the nucellus and integuments form and undergo anisotropic curvature downward and away from the developing style (Figure 1A). In the present study, ovule curvature values at specific germline stages (meiosis and early sexual ES formation) were determined and found to be less variable, likely more canalized, than ovule area values. As a result, curvature measurements were superior to area measurements in detecting differences among genotypes in onset timings of germline stages.

Meiosis and sexual ES formation occurred precociously, relative to stage of ovule development, in high AES-producing plants (Figure 5; Additional files 6, 7, 8). This was an unexpected result, and four possible explanations for its occurrence were considered. First, early onset of germline development may trigger apospory, especially in *Sorghum*, which, being a panicoid grass, may already be prone to apospory (24 Panicoideae genera contain aposporous species). However, many genotypes underwent early germline development but were not aposporous. Hence, while apospory was a good predictor of early germline development, the latter was a poor predictor of the former (Additional files 7, 8: compare ANOVA *P *and *r*^2 ^values for ovule curvature among F_2 _and RIL clustered by apospory with those obtained for frequency of apospory among F_2 _and RIL clustered by ovule curvature).

Second, meiotic instabilities due to recent hybridity may trigger apospory and early germline development. As noted above, a disproportionately high percentage of genotypes with >3% AES formation were hybridization-derived breeding lines. However, aposporous activity among the 150 genotypes tested (from 65 accessions) was not correlated with meiocyte abortion, even at *P *< 0.25. Hence, while hybridity may have increased the frequency of apospory, meiotic instability does not appear to be a factor.

Third, heterozygosity, due to recent hybridity, might trigger apospory and early germline development. If this were correct, we would expect apospory and early germline development to decline substantially during the production of the RIL population. However, apospory was present among the homozygous F_8 _RIL at nearly the same frequency (5.0% of RIL had >3.0% AES formation) as in genotypes from the accessions (7.3%) and F_2 _(7.7%). Thus, hybridity in *S. bicolor *may bring together different alleles that interact quantitatively to enhance aposporous activity, but heterozygosity does not appear to be important.

Fourth, the expression of an apomixis program in *S. bicolor*, though weak, may cause precocious reproduction, whether apomictic or sexual. This possibility best explains our observations. As noted above, apospory in a given genotype, even at the low frequencies observed herein, was a good predictor of early onset of sexual germline development. The implication is that even though the apospory program was too weak to induce consistent AES formation, it was strong enough to more consistently induce early onset of sexual germline development. While precocious aposporous and diplosporous ES formation have been documented in many apomicts [21,26-29], to our knowledge the present report is the first to document what may be a controlled heterochronic acceleration of sexual germline development by apomixis. Studies using additional sexual plants and closely related facultative apomicts are required to determine if precociousness of sexual reproduction in facultative apomicts is a general phenomenon. For such studies, curvature measurements should be useful in quantifying stages of ovule development.

Phenological traits other than ovule development also differentiate some apomicts from related sexuals. Early flowering is one. In the Netherlands, peak flowering of apomictic *Taraxacum *occurred 5 and 10 d earlier than that observed for sympatric diploid sexuals on south and north facing slopes, respectively [31]. Early flowering in apomicts was also observed among 52 apomictic and 879 sexual angiospermous species in Sweden. Here, a significantly higher proportion of apomicts (compared to the proportion of sexuals) flowered in the early spring [21]. Early flowering was also observed in natural sympatric populations of sexual and apomictic *Antennaria *Gaertn., *Boechera *Á. Löve & D. Löve, and *Elymus*. For *Antennaria*, *Boechera*, *Elymus *as well as *Tripsacum*, flowering not only occurred earlier in the apomicts but tended to continue indefinitely when grown continuously in ideal greenhouse conditions. In contrast, more specific environments were required to induce flowering in related sexuals (JGC, field collection and greenhouse notes). These examples coupled with findings presented herein, of a precocious meiosis and sexual ES formation, suggest that sexual dimorphism in plants (systematic molecular, phenological or ontogenetic differences between male, female, sexual or apomict) may be more life-cycle-pervasive than previously recognized. Sexual dimorphism at the transcriptome level (mRNA extracted from young vegetative tissues) was recently reported between male and female *Silene *L. [32].

**Figure 6:**
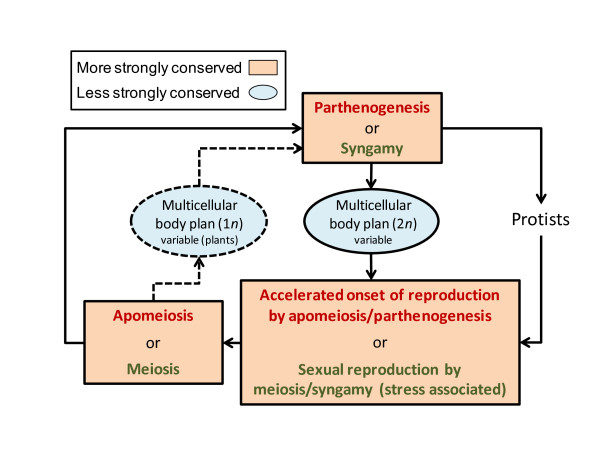
**Three reproduction decision points (rectangles) observed at temporally distinct life cycle phases during the eukaryote life cycle**. In cyclical apomicts, whether an apomictic or sexual pathway is pursued is controlled environmentally. In favorable environments, sex is suppressed and rapid reproduction by apomixis occurs. In stressful conditions, apomixis is suppressed and sex occurs (often resulting in stress-tolerant products). The two modes of reproduction require different developmental events at temporally distinct life cycle stages. An epigenomic memory of the reproductive mode during the life cycle is implicated.

The precocity of temporally distinct life-cycle events (floral induction, apomeiosis, ES formation, and parthenogenesis) may have evolved independently in apomicts. However, Asker and Jerling [21] doubted this stating that a fitness-based rationale for such directional selection at different life-cycle stages is lacking. Alternatively, the evidence to date is consistent with the existence of an apomixis program that epigenetically controls, throughout the life cycle, onset timings of temporally divergent reproduction-related events (Figure 6). In cyclically apomictic animals, e.g., certain water fleas, aphids, flatworms, rotifers, gall wasps, gall midges, and beetles, favorable environments induce a greatly accelerated rate of reproduction through apomictic live-birth parthenogenesis. But when these same individuals encounter stress, the apomixis program is suppressed, and sexual reproduction, through the formation of quiescent and stress-tolerant eggs, occurs [33]. Tendencies toward a similar cyclical apomixis in plants have been reported. Where this has been studied, percentage sexual ES formation was highest when plants were grown in suboptimal conditions (as in cyclically apomictic animals). Examples include facultative apomicts of *i*) *Boechera*, where sexual ES formation was most frequent in stressed inflorescences [34], *ii*) *Calamagrostis *Adans., where sexual ES formation was most frequent in early-forming spikelets [35], *iii*) *Ageratina *Spach [36] and *Limonium *Mill. [37], where sexual ES formation was most frequent in plants exposed to cold stress, *iv*) *Dichanthium *Willem. [38-40], where sexual ES formation was most prevalent when these short-day plants were grown in long days, and *v*) *Paspalum *L. [41] and *Brachiaria *(Trin.) Griseb. [42], where frequency of sexual ES formation was highest for plants grown in conditions unfavorable for flowering.

The hypothesis that apomixis evolves repeatedly in eukaryotes by a hybridization or polyploidization induced genetic or epigenetic uncoupling of sexual stages, where some stages are discarded and others are fortuitously retained and re-coupled [2], has received serious consideration [3-5,43]. However, a reliance on fortuity at the molecular level is a troubling component of this hypothesis, and the hypothesis in general is inconsistent with the observation that apomixis has failed to arise spontaneously (even once) among many tens of thousands of intra and inter-specific hybrids and amphiploids that have been produced artificially during the past 100 years. Herein, we suggest that the apparent uncoupling/recoupling process is not fortuitous but evidence of an ancient sex/apomixis switch (Figure 6) the molecular components of which have been retained, to a greater or lesser extent, in relatively few eukaryote lineages during evolution. Hybridization and polyploidization may occasionally epigenetically trigger the switch (from sex to apomixis or vice versa) but only in lineages that have retained, at the molecular level, a sufficient capacity for each mechanism. If this ancient alternatives hypothesis is correct, apomixis may be more complex than previously envisioned. It may be a life-cycle phenomenon, like sexual reproduction, that includes resetting the epigenetic clock each generation. Accordingly, apomixis in eukaryotes would share a common fundamental theme, i.e., the formation of unreduced and epigenetically reset parthenogenetically active cells from germline cells or closely associated cells (cells normally associated with sexual reproduction).

Similarities in the environmental control of the sex/apomixis switch between cyclically apomictic animals and facultatively apomictic plants that exhibit cyclical apomixis tendencies were recognized in the 1960s [39]. These similarities suggest that the unicellular common ancestor of plants and animals was cyclically apomictic or at least possessed processes by which cyclical apomixis could evolve by parallel evolution. In this respect, the precocious meiosis and sexual ES formation observed in the present study (Figure 5) may be regulated by the same epigenetic network that induces early flowering in apomicts, a reproductive step occurring much earlier in the life cycle, as well as precocious embryogenesis from parthenogenetic eggs [20,21], a reproductive step occurring much later in the life cycle. Molecular studies are required to evaluate these possibilities.

## Conclusions

Much variation was found among *S. bicolor *accessions in timing of germline development relative to ovary and ovule development. In this respect, ovule curvature appeared to be strongly canalized, and was more consistent than ovule area in predicting onset timing of specific germline events. AES formation was most prevalent in subsp. *verticilliflorum *and in the breeding lines of subsp. *bicolor*. It was uncommon in races of subsp. *bicolor*. Correlations between AES and AI were lower than expected, which suggests that additional factors are required for AES formation. Meiosis and sexual ES formation occurred precociously in genotypes with high AES frequencies. AES formation did not appear to be triggered by early onset of sexual germline development, meiotic instabilities or heterozygosity. Instead, a weakly expressed apomixis program in certain genotypes appeared to accelerate onset of reproduction, whether apomictic or sexual.

The present study adds onset of meiosis and sexual ES formation to onset of the vegetative/floral transition, apomictic ES formation, and parthenogenesis as processes that occur early in apomictic plants. The temporally diverse nature of these events suggests that an epigenetic memory of the apomixis status of the plant exists, which is maintained throughout the life cycle (Figure 6). In some plants, as in cyclically apomictic animals, this memory is degraded by reproductively marginal (stress-related) conditions. The result is an increased frequency of progeny that are produced sexually.

Apomictic plants share developmental and phenological traits characteristic of apomictic organisms from other kingdoms. These include *i*) a first division apomeiotic restitution (observed in many apomictic plants and animals), *ii*) parthenogenesis, *iii*) precocious onset of reproduction, and *iv*) tendencies toward cyclical apomixis. In cyclically apomictic animals and in plants exhibiting cyclical apomixis tendencies, sex is favored during stress and genetically reduced quiescent eggs are produced. In the same individuals, apomixis drives clonal fecundity during reproductively favorable conditions. The quiescent egg phase is skipped: cyclically apomictic animals, which produce quiescent eggs when reproducing sexually, undergo live birth, and the parthenogenetic eggs of apomictic plants produce embryos precociously. Whether apomicts from diverse kingdoms share molecular components of a conserved apomixis/sex switch is a question that awaits further elucidation. Such a finding would imply that apomixis is more ancient and more complex than previously envisioned.

## Methods

### Plant material

Seed of 72 *S. bicolor *accessions were obtained from the U.S. Department of Agriculture (USDA, 54 accessions), the International Crops Research Center for the Semi-arid Tropics, Hyderabad, India (ICRISAT, 4 accessions), and Boomerang Seed, Inc., Liberty Hill, TX, USA (14 breeding lines). All races of *S. bicolor *subsp. *bicolor *(bicolor, guinea, caudatum, kafir, and durra) were represented by multiple accessions. The studied plants included 21 *S. bicolor *subsp. *bicolor *breeding lines, 36 *S. bicolor *subsp. *bicolor *race or inter-race accessions, and 15 *S. bicolor *subsp. *verticilliflorum *accessions (Additional file 9). Additionally, seed of 119 F_8 _RIL were obtained from the USDA, Texas A&M University, College Station, TX, USA [30]. Parents of this RIL mapping population, BTx623 and IS3620C, were among the accessions studied (Additional file 9). Additionally, 300 genotypes of an F_2 _mapping population, produced from a single F_1_, were studied. Early Kalo (NSL 3999) was the female open-pollinated parent of the F_1_. The male parent was not identified, but molecular genotyping of Early Kalo, the F_1_, and F_2 _confirmed the hybrid status of the F_1 _(data not shown).

Seeds were sown in pots containing a 3:1:1 mixture of Sunshine Mix #1 (Sun Gro Horticulture Canada Ltd, Vancouver, BC, Canada), peat moss, and soil, respectively, and the resulting plants were grown in controlled environment greenhouses at Utah State University, Logan, UT, USA. The plants, thinned to one plant per pot, were exposed to a 32/25°C day/night temperature regime, and supplemental 1000 W high-pressure sodium-vapor lamps were used to extend the photoperiod to an 11/13 day/night photoperiod for short-day plants and a 16/8 day/night regime for day-neutral plants. A greenhouse equipped with automatic shading was used to achieve rapid flowering for short-day accessions. With supplemental lighting, daytime photosynthetic photon flux at the top of the canopy seldom fell below 600 μmol m^-2 ^sec^-1^. All plants were fertilized at each watering through an injector that delivered fertilizer (15:20:20) at approximately 250 mg L^-1^. To provide adequate samples of inflorescences of each genotype, ramets (groups of interconnected tillers) were excised from the crowns of each plant and grown as separate clones in separate pots.

### Morphometrics

Young inflorescences at the early to mid boot stage were fixed in formalin acetic acid alcohol (FAA) for 48 h and stored in 70% ethanol. Ovaries (pistils) were excised, cleared in 2:1 benzyl benzoate dibutyl phthalate, and mounted in sagittal orientation [44]. Ovaries were studied using differential interference contrast (DIC) optics of a Zeiss Universal, an Olympus BH2, and four Olympus BX53 microscopes, each equipped with digital image analysis systems. Area measurements of the entire ovule and its individual components (meiocyte or ES, nucellus, and integuments) were obtained from optical sections of sagittally oriented ovaries at the dyad to early tetrad stage, the ES1 stage, and for some plants the early ES8 stage. Ovule curvature (angle) measurements were also taken at these stages by inscribing a line from the tip of the largest inner integument of the anisotropically growing ovule to its base and then along the base of the ovule (Figure 1A). The intersecting angle was subtracted from 180, which provided a measure of the stage of ovule development (larger values corresponding to more developed ovules). Ovule area and curvature measurements were taken from 15,369 correctly staged ovules, 2820 from 116 genotypes from 57 *S. bicolor *accessions (12 to 48 ovules per stage per accession), 8328 from 300 F_2 _(generally 12 ovules per stage per F_2_), and 4221 from 119 RIL (generally 12 ovules per stage per RIL).

### Quantifying aposporous development

Frequency data for AI, AES and LSC were obtained from ramets of 569 unique genotypes (150 plants from 65 accessions, 300 F_2_, 119 RIL). Numbers of AI, AES and LSC observed were recorded for each genotype, and frequency data by genotype were determined. Numbers of ovules with a degenerating meiocyte or degenerating early sexual ES were recorded for each genotype. The percentage of ovules per accession where one or more AI, AES or LSC occurred was also determined. Frequencies were obtained by analyzing 131,727 cleared, mounted, and correctly staged (FM through ES2) ovules: 33,437 from the 65 accessions, 50,462 from the 300 F_2_, and 47,828 from the 119 RIL. Sample size per genotype ranged from 51-630 but was generally >100.

### Associating apospory with ovule morphometrics

*k*-means multivariate clustering [45] was used to cluster per-genotype AI or AES frequencies into three or four groups of similar frequency and with intra-cluster variance minimized. Three separate populations were analyzed: 115 diploid genotypes (from 57 accessions), 300 F_2 _and 119 RIL. ANOVA was used to determine if differences in ovule size (area) or curvature existed among the *k*-means groups (low to high frequency AI or AES) of each population. *k-*means groups were also determined based on ovule curvature, and ANOVA was used to determine if differences in AI or AES frequencies existed among the *k*-means groups of each of the three populations.

### Flow cytometry of nuclear DNA content

Samples (0.5 cm^2 ^each) of pre-expanded *S. bicolor *leaves were chopped for 30-60 sec using a razor blade in a few drops of Partec Extraction Buffer (CyStain UV precise P reagent kit, Partec GmbH, Münster, Germany), incubated for 2-5 min, and filtered using a Partec 50 μm CellTrics filter for each sample. Partec DAPI (4,6-diamidino-2-phenylindole) Staining Buffer (1.6 ml) was then added to each sample, and the samples were incubated for several min. Using a Partec PA flow cytometer, each sample was exposed to UV light (λ < 420 nm) and nuclear fluorescence was measured (λ = 435-500 nm). Relative fluorescence intensities from nuclei were generated and displayed using Partec software. Several diploid *S. bicolor *plants (breeding lines and races are generally diploid [12]) were used as the ploidy standard. Multiple samples were measured for each plant.

## Authors' contributions

JGC conceived of and provided the initial design of the study. MJ, EE, TNN and KKD provided important suggestions for refining the design. MJ and TNN provided cytological techniques and training, identified additional important variables to analyze, and supervised data collection. EE and JGC analyzed the data. JGC wrote the paper. All authors read and approved the final manuscript.

## Supplementary Material

Additional file 1 **Correlations between mean ovary and ovule lengths at the dyad to early tetrad (M), 1-nucleate embryo sac (ES1) and 8-nucleate embryo sac (ES8) stages of germline development**. Points represent means from 25 accessions. See Additional file 9 for accession information and Additional file 2 for sample sizes. For the regression analyses, ** and *** denote significance at *P *< 0.01 and *P *< 0.001, respectively. Click here for file

Additional file 2 **Ovary length means (±SE) at the dyad to early tetrad stage of meiosis (M_I&II_), the 1-nucleate embryo sac stage (ES1) and the early 8-nucleate embryo sac stage (ES8) for 25 accessions**. The two ANOVA main effects, accession and stage, and their interaction were highly significant (*P *< 0.001). See Additional file 9 for accession information. Numbers in bars are sample sizes. Click here for file

Additional file 3 **Frequency of aposporous initials (AI), aposporous embryo sacs (AES) and large stack cells (LSC) in ovules and ovule measurements, including mean ovule curvature (angle), ovule area in sagittal section, and percentage of ovule area in sagittal section consisting of the nucellus (NUC), the integument (INTEG), and the germ cell (meiocyte or embryo sac, GERM) for 116 *Sorghum bicolor *genotypes from 57 accessions (see Additional file **9** for accession information)**. Click here for file

Additional file 4 **Abbreviated ANOVA table for data summarized in Figure **2. Two analyses were performed, one for all 116 S. bicolor genotypes listed in Additional file 3 and one that included only genotypes from accessions of Additional file 3 represented by two or more genotypes. Click here for file

Additional file 5 **Frequency of aposporous initials (AI), aposporous embryo sacs (AES) and large stack cells (LSC) in ovules of 34 S. bicolor genotypes from 27 accessions (see Additional file **9** for accession information)**. Genotypes listed here supplement those listed in Additional file 3 for the AI, AES and LSC tally. Click here for file

Additional file 6 **Abbreviated ANOVA table for morphometric comparisons among accessions that were clustered based on frequency aposporous embryo sac (AES) formation**. The data are summarized in Figure 5A. Click here for file

Additional file 7 **Abbreviated ANOVA table for ovule curvature comparisons among F_2 _that were clustered based on frequency aposporous embryo sac (AES) formation**. The data are summarized in Figure 5B. Also listed are ANOVA F-ratios for mean AES frequency comparisons made between groups of F_2 _genotypes clustered by ovule curvature (angle) at the meiocyte (dyad through early tetrad), 1-nucleate embryo sac (ES1), and 8-nucleate embryo sac (ES8) stages. Click here for file

Additional file 8 **Abbreviated ANOVA table for ovule curvature comparisons among RIL that were clustered based on frequency aposporous initial (AI) or aposporous embryo sac (AES) formation**. The data are summarized in Figure 5C (top and bottom graphs). Also listed are ANOVA F-ratios for mean AI or AES frequency comparisons made between groups of RIL clustered by ovule curvature (angle) at the meiocyte (dyad through early tetrad) and 1-nucleate embryo sac (ES1) stages. Click here for file

Additional file 9 **Race or subspecies, common name, collection identifiers and country of origin for 72 *Sorghum bicolor *accessions evaluated for apomictic embryo sac formation and/or other morphometric variables of ovule development**. Click here for file

## References

[B1] Nogler GA Johri BM Gametophytic apomixis Embryology of Angiosperms 1984 Springer-Verlag 475 518

[B2] Carman JG Asynchronous expression of duplicate genes in angiosperms may cause apomixis, bispory, tetraspory, and polyembryony Biol J Linn Soc 1997 61 51 94 10.1111/j.1095-8312.1997.tb01778.x

[B3] Ozias-Akins P van Dik PJ Mendelian genetics of apomixis in plants Ann Rev Genet 2007 41 509 537 10.1146/annurev.genet.40.110405.090511 18076331

[B4] Tucker MR Koltunow AMG Sexual and asexual (apomictic) seed development in flowering plants: molecular, morphological and evolutionary relationships Funct Plant Biol 2009 36 490 504 10.1071/FP09078 32688664

[B5] Rodrigues JCM Koltunow AMG Epigenetic aspects of sexual and asexual seed development Acta Biol Craco Series Bot 2005 47 37 49

[B6] Charon C Moreno AB Bardou F Crespi M Non-protein-coding RNAs and their interacting RNA-binding proteins in the plant cell nucleus Mol Plant 2010 3 729 739 10.1093/mp/ssq037 20603381

[B7] Naumova TN Apomixis in angiosperms. Nucellar and Integumentary Embryony 1993 Boca Raton, CRC Press

[B8] APG III An update of the angiosperm phylogeny group classification for the orders and families of flowering plants Biol J Linn Soc 2009 161 105 121 10.1111/j.1095-8339.2009.00996.x

[B9] Abrouk M Murat F Pont C Messing J Jackson S Faraut T Tannier E Plomion C Cooke R Feuillet C Salse J Palaeogenomics of plants: synteny-based modeling of extinct ancestors Trends Plant Sci 2010 15 479 487 10.1016/j.tplants.2010.06.001 20638891

[B10] Paterson AH Bowers JE Bruggmann R Dubchak I Grimwood J Gundlach H Haberer G Hellsten U Mitros T Poliakov A Schmutz J Spannagl M Tang H Wang X Wicker T Bharti AK Chapman J Feltus FA Gowik U Grigoriev IV Lyons E Maher CA Martis M Narechania A Otillar RP Penning BW Salamov AA Wang Y Zhang L Carpita NC Freeling M Gingle AR Hash CT Keller B Klein P Kresovich S McCann MC Ming R Peterson DG Mehboob-ur-Rahman Ware D Westhoff P Mayer KFX Messing J Rokhsar DS The *Sorghum bicolor *genome and the diversification of grasses Nature 2009 457 551 556 10.1038/nature07723 19189423

[B11] Van de Peer Y Maere S Meyer A The evolutionary significance of ancient genome duplications Nat Rev Genet 2009 10 725 732 10.1038/nrg2600 19652647

[B12] Dahlberg JA Smith CW, Frederiksen RA Classification and characterization of sorghum Sorghum: Origin, History, Technology, and Production 2000 John Wiley & Sons 99 130

[B13] Hanna WW Schertz KF Bashaw EC Apospory in *Sorghum bicolor *(L.) Moench Science 1970 170 338 339 10.1126/science.170.3955.338 17731307

[B14] Reddy CS Schertz KF Bashaw EC Apomictic frequency in sorghum R473 Euphytica 1980 29 223 226 10.1007/BF00025118

[B15] Tang CY Schertz KF Bashaw EC Apomixis in sorghum lines and their F1 progenies Bot Gaz 1980 141 294 299 10.1086/337159

[B16] Rana BS Reddy CS Rao VJM Rao NGP Apomixis in grain sorghums: analysis of seed set and effects of selection Indian J Genet Plant Breed 1981 41 118 123

[B17] Elkonin LA Enaleeva N-Kh Tsvetova MI Belyaeva EV Ishin AG Partially fertile line with apospory obtained from tissue culture of male sterile plant of sorghum (*Sorghum bicolor *L. Moench) Ann Bot 1995 76 359 364 10.1006/anbo.1995.1108

[B18] Marshall DR Downes RW A test for obligate apomixis in grain sorghum R473 Euphytica 1977 26 661 664 10.1007/BF00021691

[B19] Bala Ravi S Apomixis in sorghum line R473 - truth or myth? A critical analysis of published work Curr Sci 1993 64 306 315

[B20] Gustafsson Å Apomixis in higher plants. I. The mechanism of apomixis Lunds Univ Årssk 1946 42 1 67

[B21] Asker SE Jerling L Apomixis in Plants 1992 Boca Raton: CRC Press

[B22] Sánchez-Ken JG Clark LG Phylogeny and a new tribal classification of the Panicoideae S.L. (Poaceae) based on plastid and nuclear sequence data and structural data Amer J Bot 2010 97 1732 1748 10.3732/ajb.1000024 21616806

[B23] Crane DF Savidan Y, Carman JG, Dresselhaus T Classification of apomictic mechanisms The Flowering of Apomixis: From Mechanisms to Genetic Engineering 2001 Mexico, D.F.: CIMMYT, IRD, European Commission DG VI (FAIR) 24 43

[B24] Sherwood RT Savidan Y, Carman JG, Dresselhaus T Genetic analysis of apomixis The Flowering of Apomixis: From Mechanisms to Genetic Engineering 2001 Mexico, D.F.: CIMMYT, IRD, European Commission DG VI (FAIR) 64 82

[B25] Matzk F Meister A Schubert I An efficient screen for reproductive pathways using mature seeds of monocots and dicots Plant J 2000 21 97 108 10.1046/j.1365-313x.2000.00647.x 10652155

[B26] Leblanc O Savidan Y Timing of megasporogenesis in *Tripsacum *species (Poaceae) as related to the control of apomixis and sexuality Pol Bot Stud 1994 8 75 81

[B27] Peel MD Carman JG Leblanc O Megasporocyte callose in apomictic buffelgrass, Kentucky bluegrass, *Pennisetum squamulatum *Fresen, *Tripsacum *L. and weeping lovegrass Crop Sci 1997 37 724 732 10.2135/cropsci1997.0011183X003700030006x

[B28] Peel MD Carman JG Liu ZW Wang RRC Meiotic anomalies in hybrids between wheat and apomictic *Elymus rectisetus *(Nees in Lehm.) A. Löve & Connor Crop Sci 1997 37 717 723 10.2135/cropsci1997.0011183X003700030005x

[B29] Carman JG Hörandl E, Grossniklaus U, van Dijk PJ, Sharbel TF Do duplicate genes cause apomixis? Apomixis: evolution, mechanisms and perspectives 2007 ARG Gantner Verlag KG 63 91

[B30] Menz MA Klein RR Mullet JE Obert JA Unruh NC Klein PE A high-density genetic map of *Sorghum bicolor *(L.) Moench based on 2926 AFLP^®^, RFLP and SSR markers Plant Mol Biol 2002 48 483 499 10.1023/A:1014831302392 11999830

[B31] Verduijn VH Van Dijk PJ Van Damme JMM Distribution, phenology and demography of sympatric sexual and asexual dandelions (*Taraxacum officinale *s.l.): geographic parthenogenesis on a small scale Biol J Linn Soc 2004 82 205 218 10.1111/j.1095-8312.2004.00325.x

[B32] Zluvova J Zak J Janousek B Vyskot B Dioecious *Silene latifolia *plants show sexual dimorphism in the vegetative stage BMC Plant Biol 2010 10 208 10.1186/1471-2229-10-208 20854681 PMC2956557

[B33] Suomalainen E Saura A Lokki J Cytology and Evolution in Parthenogenesis 1987 CRC Press, Baca Raton

[B34] Böcher TW Cytological and embryological studies in the amphi-apomictic *Arabis holboellii-*complex Danske Viden Selskab, Biol Skrifter 1951 6 1 59

[B35] Nygren A Form and biotype formation in *Calamagrostis purpurea* Hereditas 1951 37 519 532 10.1111/j.1601-5223.1951.tb02908.x

[B36] Sparvioli E Osservazioni cito-embryologiche in *Eupatorium riparium *Reg. II. Megasporogenesi e sviluppo del gametofito femminile Ann di Bot 1960 26 481 504

[B37] Hjelmqvist H Grazi F Studies on variation in embryo sac development Bot Not 1964 117 141 166

[B38] Knox RB Heslop-Harrison J Experimental control of aposporous apomixis in a grass of the Andropogoneae Bot Not 1963 116 127 141

[B39] Knox RB Apomixis: seasonal and population differences in a grass Science 1967 157 325 326 10.1126/science.157.3786.325 6028403

[B40] Saran S de Wet JMJ Environmental control of reproduction in *Dichanthium intermedium* J Cyto Genet 1976 11 22 28

[B41] Quarin CL Seasonal changes in the incidence of apomixis of diploid, triploid, and tetraploid plants of *Paspalum cromyorrhizon* Euphytica 1986 35 515 522 10.1007/BF00021860

[B42] Lutts S Ndikumana J Louant BP Male and female sporogenesis and gametogenesis in apomictic *Brachiaria brizantha, Brachiaria decumbens *and F_1 _hybrids with sexual colchicine induced tetraploid *Brachiaria ruziziensis* Euphytica 1994 78 19 25

[B43] Sharbel TF Voigt M-L Corral JM Galla G Kumlehn J Klukas C Schreiber F Vogel H Rotter B Apomictic and sexual ovules of *Boechera *display heterochronic global gene expression patterns Plant Cell 2010 655 671 10.1105/tpc.109.072223 20305122 PMC2861462

[B44] Crane CF Carman JG Mechanisms of apomixis in *Elymus rectisetus *from Eastern Australia and New Zealand Amer J Bot 74 477 496 10.2307/2443827

[B45] SYSTAT SYSTAT for Windows SYSTAT Software, Inc 2004 Ver. No. 11.00.01

