# Supplementary figures and images for: Apospory appears to accelerate onset of meiosis and sexual embryo sac formation in sorghum ovules
Source: BMC Plant Biol. 2011 Jan 11;11:9. doi: 10.1186/1471-2229-11-9 (PMC3023736; doi:10.1186/1471-2229-11-9)

**Additional file 1**

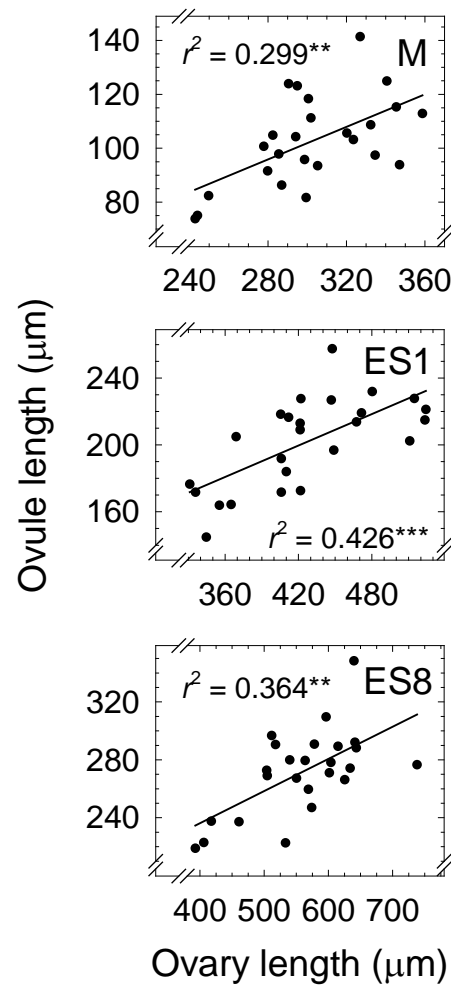

Supplement: Additional file 1 — Correlations between mean ovary and ovule lengths at the dyad to early tetrad (M), 1-nucleate embryo sac (ES1) and 8-nucleate embryo sac (ES8) stages of germline development. Points represent means from 25 accessions. See Additional file 9 for accession information and Additional file 2 for sample sizes. For the regression analyses, ** and *** denote significance at P < 0.01 and P < 0.001, respectively. [file 1471-2229-11-9-S1.PDF]

## Additional file 2

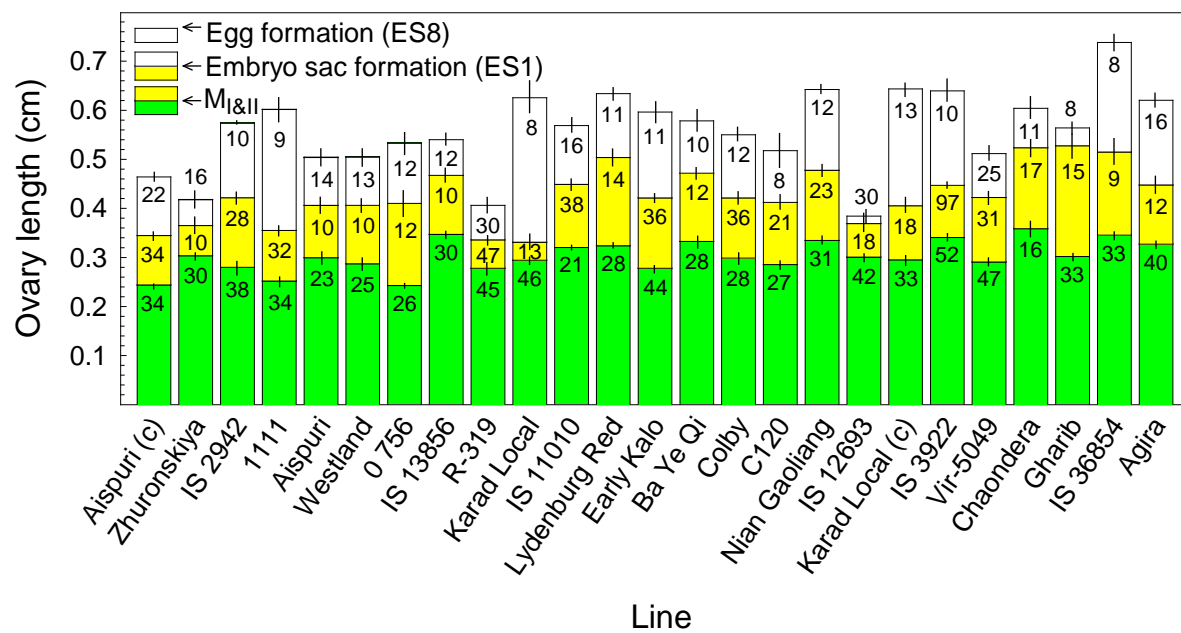

Supplement: Additional file 2 — Ovary length means (±SE) at the dyad to early tetrad stage of meiosis (MI&II), the 1-nucleate embryo sac stage (ES1) and the early 8-nucleate embryo sac stage (ES8) for 25 accessions. The two ANOVA main effects, accession and stage, and their interaction were highly significant (P < 0.001). See Additional file 9 for accession information. Numbers in bars are sample sizes. [file 1471-2229-11-9-S2.PDF]
