# Supplementary material for: Apospory appears to accelerate onset of meiosis and sexual embryo sac formation in sorghum ovules
Source: BMC Plant Biol. 2011 Jan 11;11:9. doi: 10.1186/1471-2229-11-9 (PMC3023736; doi:10.1186/1471-2229-11-9)
Supplement: Additional file 3 — Frequency of aposporous initials (AI), aposporous embryo sacs (AES) and large stack cells (LSC) in ovules and ovule measurements, including mean ovule curvature (angle), ovule area in sagittal section, and percentage of ovule area in sagittal section consisting of the nucellus (NUC), the integument (INTEG), and the germ cell (meiocyte or embryo sac, GERM) for 116 Sorghum bicolor genotypes from 57 accessions (see Additional file 9 for accession information). [file 1471-2229-11-9-S3.PDF]

Additional file 3

Taxonomic description	Accession common name	Geno-type	n	Frequency (%)			Stage	Mean ± SE				
				AI	AES	LSC		Angle	Area (μm^2)	NUC %	INTEG %	GERM %
Race (subspecies *bicolor*)												
bicolor	4528	79a	163	0.00	0.61	0.61	Meiocyte	126.6 ± 1.8	15528 ± 730	59.6 ± 0.6	36.8 ± 0.6	3.7 ± 0.23
							ES1	142.5 ± 2.3	29346 ± 2711	65.7 ± 0.6	32.8 ± 0.6	1.4 ± 0.16
		79b	241	0.00	0.41	1.24	Meiocyte	125 ± 2.1	11217 ± 543	58.3 ± 0.7	36.8 ± 0.7	5 ± 0.28
							ES1	153.2 ± 1.2	45187 ± 1480	67.9 ± 0.4	30.8 ± 0.3	1.4 ± 0.12
	Mukaruki	80a	379	0.00	0.00	0.26	Meiocyte	122.3 ± 2.1	10183 ± 547	54 ± 0.4	40.8 ± 0.5	5.2 ± 0.31
							ES1	145 ± 1.1	31821 ± 1602	56.1 ± 1	42.3 ± 0.9	1.6 ± 0.13
		80b	142	0.00	0.00	0.00	Meiocyte	129.9 ± 1.3	11891 ± 707	59.1 ± 0.4	35.5 ± 0.6	5.4 ± 0.3
							ES1	149.3 ± 0.8	26680 ± 545	60.6 ± 0.3	37.6 ± 0.4	1.8 ± 0.09
		80c	174	1.72	0.00	1.72	Meiocyte	132.5 ± 1.5	11746 ± 704	56.1 ± 0.4	38.4 ± 0.4	5.5 ± 0.29
							ES1	147.8 ± 1.7	27517 ± 1868	61.6 ± 0.3	36.5 ± 0.4	1.9 ± 0.15
	Farfara	84a	234	0.00	0.00	0.85	Meiocyte	124.9 ± 1.3	11527 ± 830	65.5 ± 1.1	29.9 ± 1.1	4.6 ± 0.19
							ES1	149.3 ± 1.5	31617 ± 1503	70.7 ± 0.9	27.5 ± 0.8	1.9 ± 0.17
		84b	440	0.23	0.00	3.41	Meiocyte	134.5 ± 1.2	11218 ± 436	58.7 ± 0.4	36.8 ± 0.3	4.5 ± 0.19
							ES1	153.6 ± 0.5	33079 ± 964	64.9 ± 0.3	33.7 ± 0.3	1.4 ± 0.1
		84c	303	1.98	0.66	3.30	Meiocyte	120.8 ± 2.1	11767 ± 641	64.1 ± 0.4	32 ± 0.4	3.9 ± 0.2
							ES1	147 ± 1.6	34856 ± 1520	70.3 ± 1	28.4 ± 1.1	1.3 ± 0.13
	410	85c	297	3.37	0.00	5.39	Meiocyte	127.6 ± 2.3	12633 ± 396	59.7 ± 1.1	36.3 ± 1.2	4 ± 0.21
							ES1	144.8 ± 1.9	28650 ± 1510	63.8 ± 0.5	35.2 ± 0.6	1 ± 0.11
caudatum	Ba Ye Qi	13.1a	166	6.02	0.60	19.88	Meiocyte	127 ± 1.7	11228 ± 694	65.6 ± 0.6	28.7 ± 0.8	5.7 ± 0.35
							ES1	141.1 ± 1.2	22349 ± 1144	68.2 ± 0.3	30 ± 0.4	1.9 ± 0.15
		13.1c	186	3.76	2.69	26.88	Meiocyte	125.7 ± 1.8	12046 ± 809	64.5 ± 0.6	30.5 ± 0.4	5 ± 0.35
							ES1	145 ± 1.7	29758 ± 1936	68.5 ± 0.6	30.1 ± 0.6	1.4 ± 0.14
	Nian Gaoliang	13.2a	250	3.20	3.60	10.80	Meiocyte	130.3 ± 1.3	13259 ± 737	63 ± 0.4	32.3 ± 0.4	4.8 ± 0.26
							ES1	143.7 ± 1.8	30037 ± 3117	67.5 ± 0.5	31 ± 0.6	1.6 ± 0.12
		13.2b	205	0.98	0.00	3.41	Meiocyte	133.5 ± 1.3	15280 ± 592	63.9 ± 0.9	32.3 ± 0.9	3.8 ± 0.28

							ES1	149.4 ± 1.6	29373 ± 1718	67.5 ± 0.4	31.2 ± 0.5	1.4 ± 0.09
		13.2c	494	2.43	1.62	21.05	Meiocyte	128.8 ± 1.7	11727 ± 736	65.1 ± 0.5	29.7 ± 0.6	5.2 ± 0.34
							ES1	148.7 ± 1.1	30499 ± 1443	69.5 ± 0.4	29 ± 0.4	1.5 ± 0.1
	Agira	9.2a	210	0.00	0.00	1.43	Meiocyte	138.9 ± 2.1	14410 ± 749	57.9 ± 0.8	38 ± 0.6	4 ± 0.26
							ES1	157.1 ± 0.9	39891 ± 2885	68.6 ± 0.5	30 ± 0.6	1.8 ± 0.24
		9.2b	198	3.54	0.51	3.03	Meiocyte	141.3 ± 2.8	12350 ± 564	55.2 ± 1.8	40.8 ± 1.7	4 ± 0.27
							ES1	155.7 ± 1.1	39598 ± 1753	67.9 ± 0.4	30.7 ± 0.5	1.4 ± 0.17
		9.2c	98	1.02	0.00	8.16	Meiocyte	138.1 ± 2.3	11914 ± 655	57.3 ± 1	38.1 ± 1.1	4.6 ± 0.22
							ES1	154.8 ± 1.8	42566 ± 2666	69.4 ± 0.6	29.6 ± 0.6	1 ± 0.13
	015A	98b	292	0.68	0.68	3.77	Meiocyte	133.6 ± 1.5	11940 ± 729	61.7 ± 0.4	34 ± 0.6	4.2 ± 0.26
							ES1	149 ± 1.3	34366 ± 2050	66.1 ± 0.5	32.7 ± 0.5	1.2 ± 0.1
	IS 11888	99b	152	0.00	0.00	1.97	Meiocyte	115.2 ± 2	14374 ± 1208	63.3 ± 0.3	33.4 ± 0.4	3.3 ± 0.42
							ES1	144.5 ± 1.6	33783 ± 2451	69.7 ± 1.6	28.6 ± 1.8	1.2 ± 0.1
durra	Aispuri-C	1.2a	453	1.10	0.44	9.93	Meiocyte	128.1 ± 3.9	13425 ± 1533	67.3 ± 1.7	28.4 ± 1.6	4.2 ± 0.32
							ES1	148.1 ± 1.9	37353 ± 1966	72 ± 1.2	26.4 ± 1.1	1.5 ± 0.22
		1.2b	195	0.51	0.51	2.05	Meiocyte	124.3 ± 4.1	11739 ± 779	62.3 ± 1.8	32.9 ± 1.7	4.8 ± 0.33
							ES1	138.4 ± 1.8	28811 ± 2258	65.6 ± 0.9	32.4 ± 0.9	2 ± 0.09
		1.2c	229	0.00	0.00	0.87	Meiocyte	115.3 ± 2.2	11318 ± 540	63.7 ± 0.6	31.6 ± 0.6	4.8 ± 0.17
							ES1	130.4 ± 2.3	19927 ± 1672	66.5 ± 0.7	31.2 ± 0.7	2.4 ± 0.34
	IS 13856	10.2a	293	0.00	0.00	0.34	Meiocyte	139.5 ± 1.6	12434 ± 353	48.7 ± 0.7	46.9 ± 0.6	4.3 ± 0.19
							ES1	153.2 ± 1.7	32020 ± 1676	55.5 ± 0.9	43.1 ± 0.9	1.4 ± 0.08
	Chaondera	11.1a	286	0.70	1.05	3.50	Meiocyte	132.7 ± 1.9	11530 ± 904	36.7 ± 7.6	58.1 ± 7.3	5.3 ± 0.36
							ES1	146.1 ± 1.7	21015 ± 1432	57 ± 1.1	40.7 ± 1	2.3 ± 0.28
	IS 36854	12.1a	142	0.00	0.00	2.82	Meiocyte	134.8 ± 1.5	15031 ± 1030	62.7 ± 0.7	32.7 ± 0.6	4.6 ± 0.27
							ES1	148.1 ± 1.1	33885 ± 2155	67 ± 0.8	31.6 ± 0.8	1.4 ± 0.09
		12.1b	269	0.37	0.37	7.06	Meiocyte	128.2 ± 2.2	16986 ± 1234	66.7 ± 1.3	29.4 ± 1.4	3.8 ± 0.29
							ES1	146.2 ± 1.6	28534 ± 1682	70.1 ± 0.5	28.9 ± 0.5	0.9 ± 0.12
	Gharib	12.2a	311	4.50	1.61	24.44	Meiocyte	132 ± 3.3	14822 ± 966	60.9 ± 2.3	34.7 ± 2.5	4.4 ± 0.36
							ES1	144.7 ± 1.6	30354 ± 2705	69.9 ± 0.6	29 ± 0.6	1.1 ± 0.1

		12.2b	230	1.30	2.17	3.91	Meiocyte	124.9 ± 1.1	10809 ± 565	63.9 ± 0.4	31 ± 0.4	5.1 ± 0.31
							ES1	145.2 ± 1.9	30562 ± 2549	68.2 ± 0.5	30.5 ± 0.5	1.3 ± 0.12
	Karad Local-C	4.1a	445	0.67	0.67	12.36	Meiocyte	128.3 ± 1.8	11546 ± 576	67.9 ± 0.8	27.6 ± 0.8	4.5 ± 0.23
							ES1	146.8 ± 2.4	34563 ± 2204	71.3 ± 0.9	27.4 ± 1	1.2 ± 0.11
		4.1b	383	1.31	0.00	7.83	Meiocyte	130.8 ± 1.8	12553 ± 409	67 ± 0.4	28.8 ± 0.5	4.2 ± 0.16
							ES1	148.8 ± 2	31059 ± 1803	73.1 ± 0.6	25.7 ± 0.6	1.1 ± 0.11
		4.1c	241	0.00	0.00	4.56	Meiocyte	122.7 ± 2.1	11661 ± 900	66.2 ± 0.7	28.8 ± 0.8	5 ± 0.2
							ES1	141.3 ± 1.5	25042 ± 2186	72.8 ± 0.4	26.1 ± 0.5	1.1 ± 0.06
	Colby	6.1a	365	0.27	0.55	0.82	Meiocyte	123.7 ± 1.8	13174 ± 581	60.9 ± 0.5	33.8 ± 0.6	5.3 ± 0.31
							ES1	149.6 ± 1.8	37504 ± 2629	67.8 ± 0.6	30.7 ± 0.7	1.3 ± 0.11
		6.1b	191	0.52	0.00	1.57	Meiocyte	124.9 ± 2	13864 ± 870	59.3 ± 0.6	36.8 ± 0.7	3.9 ± 0.33
							ES1	150.3 ± 0.7	38554 ± 1337	64.5 ± 0.6	34.4 ± 0.6	1.2 ± 0.11
		6.1e	144	2.08	0.69	1.39	Meiocyte	126 ± 2.1	12956 ± 597	61.1 ± 0.6	33.8 ± 0.6	5.1 ± 0.22
							ES1	144 ± 1.7	29549 ± 2266	68.5 ± 0.6	29.9 ± 0.6	1.6 ± 0.19
	Vir-5049	9.1a	196	0.51	0.00	1.02	Meiocyte	136.9 ± 3.1	15431 ± 1001	55.4 ± 0.8	40.9 ± 0.9	3.7 ± 0.31
							ES1	149.4 ± 0.8	31385 ± 1525	65.4 ± 1.1	33.2 ± 1.1	1.4 ± 0.11
		9.1c	277	0.00	0.36	0.36	Meiocyte	128.3 ± 2.4	14160 ± 1178	60 ± 0.7	35.3 ± 0.8	4.6 ± 0.33
							ES1	148.3 ± 1.3	40965 ± 2917	68.7 ± 0.8	30.1 ± 0.7	1.2 ± 0.11
guinea	Baba Founfoun	87a	199	2.01	0.50	2.51	Meiocyte	128 ± 1.6	11782 ± 875	56.2 ± 0.9	39.4 ± 0.9	4.4 ± 0.29
							ES1	146.3 ± 1.3	32988 ± 2233	62.2 ± 0.8	36.1 ± 0.8	1.7 ± 0.14
	Keninke Teli	88a	165	0.61	0.00	3.03	Meiocyte	122.1 ± 2	12513 ± 1092	60.6 ± 0.6	34.9 ± 0.6	4.5 ± 0.23
							ES1	145.8 ± 1.2	30661 ± 1894	65.1 ± 0.9	33.8 ± 0.8	1.2 ± 0.17
		88b	298	1.34	0.00	1.01	Meiocyte	121.3 ± 1.8	12113 ± 561	61.6 ± 0.6	34.1 ± 0.7	4.3 ± 0.26
							ES1	144.7 ± 1.5	33158 ± 2570	67.7 ± 0.6	31.3 ± 0.5	1 ± 0.08
		88c	177	0.00	0.00	5.08	Meiocyte	122.3 ± 2.9	13957 ± 637	60.8 ± 0.4	35.6 ± 0.4	3.6 ± 0.18
							ES1	139.3 ± 2	28660 ± 2613	65.1 ± 0.7	34.1 ± 0.7	0.9 ± 0.09
	Sokombe	89b	274	0.73	0.36	2.92	Meiocyte	131.3 ± 2.3	11537 ± 452	50.9 ± 0.4	44.6 ± 0.5	4.5 ± 0.22
							ES1	150.8 ± 0.9	33470 ± 1157	60.2 ± 0.2	38.5 ± 0.3	1.3 ± 0.09
		89c	78	0.00	0.00	0.00	Meiocyte	140.5 ± 1.4	14817 ± 542	52.2 ± 0.5	44.3 ± 0.4	3.5 ± 0.2

							ES1	154.3 ± 0.7	32868 ± 2178	60.6 ± 0.9	38.3 ± 0.9	1.1 ± 0.11
	Bassi Wende	90a	355	2.54	0.00	5.63	Meiocyte	130.3 ± 1.4	12459 ± 681	58.1 ± 0.5	37.9 ± 0.5	4.1 ± 0.31
							ES1	146.5 ± 2.5	31634 ± 3115	64 ± 0.7	34.8 ± 0.6	1.3 ± 0.13
	Tchari	91a	283	4.59	1.06	19.08	Meiocyte	125.6 ± 2.1	11505 ± 774	60.7 ± 0.8	33.8 ± 0.9	5.5 ± 0.29
							ES1	145.5 ± 1.2	29397 ± 1343	67.4 ± 0.5	30.9 ± 0.5	1.7 ± 0.11
kafir	Lydenburg Red	10.1a	334	1.80	0.30	5.39	Meiocyte	121.7 ± 2.7	13546 ± 971	62.3 ± 0.7	33.3 ± 0.8	4.4 ± 0.27
							ES1	140 ± 1.5	26769 ± 1657	68 ± 0.7	30.8 ± 0.7	1.2 ± 0.1
		10.1b	251	4.78	0.80	12.35	Meiocyte	124.5 ± 2.1	12820 ± 795	60.6 ± 0.8	34.7 ± 0.8	4.7 ± 0.36
							ES1	144.8 ± 0.8	30853 ± 1186	65.1 ± 0.6	33.5 ± 0.5	1.3 ± 0.11
		10.1e	119	3.36	2.52	6.72	Meiocyte	120.7 ± 2.3	11176 ± 514	60.4 ± 0.6	34.8 ± 0.6	4.8 ± 0.15
							ES1	144.2 ± 1.2	29003 ± 1794	63.4 ± 0.5	35.3 ± 0.5	1.3 ± 0.14
	IS 2942	2.1c	372	2.69	0.00	4.03	Meiocyte	126.1 ± 1.6	8941 ± 509	56.6 ± 0.7	37.2 ± 0.7	6.2 ± 0.44
							ES1	146.2 ± 1.7	20879 ± 1330	61.8 ± 0.7	36 ± 0.6	2.2 ± 0.24
	IS 3922	3.1a	332	0.60	0.00	0.60	Meiocyte	133.9 ± 1.3	13797 ± 597	59.6 ± 0.8	36.7 ± 0.9	3.7 ± 0.32
							ES1	148.3 ± 1.2	36581 ± 1507	69.5 ± 0.7	29.2 ± 0.7	1.2 ± 0.13
		3.1d	292	0.34	0.00	1.03	Meiocyte	131.9 ± 1.2	13285 ± 657	59.6 ± 0.8	35.6 ± 0.8	4.9 ± 0.34
							ES1	149.2 ± 1.1	30162 ± 2282	67.3 ± 0.9	31.4 ± 0.8	1.4 ± 0.09
		3.1e	247	0.81	0.40	8.91	Meiocyte	129.8 ± 2.1	12818 ± 687	61.9 ± 1	33.4 ± 1.1	4.8 ± 0.31
							ES1	145.5 ± 1.2	33614 ± 1878	67.8 ± 0.6	30.8 ± 0.6	1.4 ± 0.11
	IS 28865	92a	179	0.00	0.56	1.12	Meiocyte	129.5 ± 2.3	13635 ± 520	60.9 ± 0.5	34.6 ± 0.6	4.4 ± 0.27
							ES1	139.9 ± 1.7	20025 ± 1221	63.5 ± 0.5	35.2 ± 0.5	1.4 ± 0.07
	Dewe	93a	254	2.76	0.79	12.20	Meiocyte	124.2 ± 1.2	12185 ± 268	60.8 ± 0.4	34.9 ± 0.5	4.4 ± 0.21
							ES1	143.9 ± 1.6	25990 ± 1045	62.6 ± 0.5	36 ± 0.5	1.5 ± 0.09
		93b	298	1.34	0.34	1.68	Meiocyte	119.3 ± 1.3	12876 ± 576	62.4 ± 0.6	33.1 ± 0.7	4.5 ± 0.31
							ES1	143.9 ± 0.8	33758 ± 1461	65.5 ± 0.5	33.3 ± 0.5	1.2 ± 0.05
		93d	215	1.40	0.00	0.93	Meiocyte	121.2 ± 0.9	12517 ± 337	61.6 ± 0.5	33.8 ± 0.5	4.6 ± 0.19
							ES1	140.2 ± 1.4	25716 ± 1160	62.1 ± 0.3	36.4 ± 0.4	1.6 ± 0.09
	White Kafir	94a	327	1.22	0.31	3.06	Meiocyte	116.1 ± 1.1	11575 ± 784	67.3 ± 0.7	27.9 ± 0.6	4.9 ± 0.22
							ES1	134.7 ± 1.2	25525 ± 1942	70.6 ± 0.6	27.6 ± 0.5	1.8 ± 0.15

		94b	390	0.26	0.26	3.08	Meiocyte	116.8 ± 2.4	11850 ± 698	66.2 ± 0.7	28.8 ± 0.7	5 ± 0.37
							ES1	133.5 ± 1.9	24498 ± 926	70.6 ± 0.8	27.5 ± 0.8	1.9 ± 0.14
		94c	344	1.16	0.29	8.43	Meiocyte	115.2 ± 1.1	11023 ± 529	68.7 ± 0.6	25.9 ± 0.6	5.4 ± 0.22
							ES1	137.5 ± 2.3	31570 ± 1721	73.2 ± 0.8	25.3 ± 0.7	1.5 ± 0.08
	Combine Kafir	95a	184	0.00	0.00	2.17	Meiocyte	121.4 ± 1.3	12716 ± 576	62.5 ± 0.6	32.4 ± 0.7	5.1 ± 0.19
							ES1	141.2 ± 2	27276 ± 1168	65.8 ± 0.6	32.5 ± 0.6	1.6 ± 0.1
		95b	334	0.30	0.00	0.90	Meiocyte	118.2 ± 2.2	11635 ± 462	61.7 ± 0.7	33.1 ± 0.6	5.2 ± 0.32
							ES1	140.7 ± 1.6	30811 ± 1618	65 ± 0.8	32.9 ± 0.9	2.1 ± 0.15
durra-caudatum	Early Kalo	5.1a	135	5.19	0.74	0.00	Meiocyte	122 ± 2.2	11705 ± 865	60.3 ± 1	35.2 ± 1.1	4.4 ± 0.26
							ES1	140.6 ± 2.1	28023 ± 1482	62 ± 0.8	36.8 ± 0.8	1.2 ± 0.12
		5.1q	216	3.70	1.39	0.46	Meiocyte	129.4 ± 2.2	12694 ± 569	57.8 ± 0.8	37.7 ± 1	4.6 ± 0.23
							ES1	144.1 ± 0.9	27588 ± 702	62.3 ± 0.5	36.6 ± 0.4	1.1 ± 0.1
		5.1t	180	0.00	0.00	0.56	Meiocyte	127.5 ± 1.7	13934 ± 629	59.7 ± 0.7	36.2 ± 0.7	4.1 ± 0.32
							ES1	144.6 ± 2	28448 ± 1384	63.3 ± 0.7	35.2 ± 0.7	1.5 ± 0.11
		5.1u	146	0.00	0.68	0.68	Meiocyte	128 ± 2.8	13372 ± 819	58 ± 1	37.4 ± 1.3	4.6 ± 0.46
							ES1	147.3 ± 0.7	30191 ± 983	62.7 ± 0.6	36.2 ± 0.6	1.1 ± 0.15
	Westland	5.2a	226	0.44	0.00	0.44	Meiocyte	124.4 ± 2.9	14270 ± 615	57.5 ± 0.6	37.7 ± 0.5	4.8 ± 0.3
							ES1	142 ± 1.2	27851 ± 1595	64.4 ± 0.7	33.9 ± 0.7	1.6 ± 0.13
		5.2c	344	0.87	0.00	0.29	Meiocyte	118.5 ± 2.5	13388 ± 602	59.4 ± 0.8	36 ± 0.8	4.6 ± 0.26
							ES1	139.4 ± 1.6	29581 ± 1702	65.7 ± 0.5	32.9 ± 0.5	1.4 ± 0.11
breeding line	TX2737	128a	268	2.24	0.75	5.97	Meiocyte	131.5 ± 1.4	14140 ± 770	61.2 ± 0.6	34.6 ± 0.6	4.3 ± 0.31
							ES1	152.7 ± 1.4	42622 ± 1772	69.3 ± 0.7	29.8 ± 0.7	1 ± 0.09
	Combine Shallu	17a	179	1.12	0.00	0.56	Meiocyte	135 ± 1.5	13508 ± 587	58.3 ± 0.7	37.6 ± 0.6	4.1 ± 0.42
							ES1	145.8 ± 1.6	28767 ± 2207	66 ± 0.9	32.8 ± 1	1.2 ± 0.11
	IS 3620C	19a	219	7.76	5.02	33.79	Meiocyte	118.4 ± 2.2	11245 ± 456	53.3 ± 0.7	41.8 ± 0.9	4.9 ± 0.28
							ES1	145.1 ± 1.6	28061 ± 1776	58.7 ± 1	40 ± 1	1.3 ± 0.08
		19b	421	8.31	3.56	34.44	Meiocyte	120.9 ± 2.2	9716 ± 382	50.5 ± 0.7	44.7 ± 0.8	4.8 ± 0.35
							ES1	142.8 ± 2	26029 ± 1489	59.1 ± 0.5	39.2 ± 0.5	1.7 ± 0.13

		19c	346	8.09	5.78	32.95	Meiocyte	120.3 ± 1.4	10168 ± 249	54.9 ± 0.5	40.3 ± 0.6	4.9 ± 0.26
							ES1	131.9 ± 1.6	19242 ± 1203	59.9 ± 0.8	38.5 ± 0.8	1.6 ± 0.14
	ARG1	31a	293	3.75	0.34	1.37	Meiocyte	131.2 ± 0.8	11943 ± 543	59 ± 0.6	36.5 ± 0.8	4.5 ± 0.35
							ES1	147.6 ± 1.6	30303 ± 1138	62.9 ± 0.4	35.7 ± 0.5	1.4 ± 0.07
	B.TX642	34a	241	0.83	0.00	2.90	Meiocyte	124.7 ± 1.9	10917 ± 274	49.6 ± 5	45.7 ± 5	4.7 ± 0.21
							ES1	146.3 ± 2.4	23656 ± 1633	64.9 ± 0.7	32.9 ± 0.6	2.3 ± 0.18
	TX3042	42a	194	1.03	0.52	1.55	Meiocyte	128.8 ± 2.1	13688 ± 612	64.1 ± 0.7	31.9 ± 0.7	4 ± 0.28
							ES1	152.5 ± 1.1	36921 ± 2358	68.1 ± 0.4	30.8 ± 0.5	1.1 ± 0.09
	QL36	43a	238	4.20	3.36	1.68	Meiocyte	120.5 ± 2.8	11601 ± 570	62.2 ± 1	34 ± 1.1	3.8 ± 0.31
							ES1	135.9 ± 1.7	28969 ± 1974	67.5 ± 1.6	30.9 ± 1.5	1.7 ± 0.15
	TX414	65a	176	0.00	0.57	1.70	Meiocyte	125.8 ± 2.1	13711 ± 724	60.2 ± 0.8	35.2 ± 0.8	4.6 ± 0.29
							ES1	145 ± 2.2	30927 ± 3022	65.2 ± 0.9	33.4 ± 0.9	1.4 ± 0.15
	TX2741	67a	181	2.21	1.10	1.10	Meiocyte	136.9 ± 1.2	17091 ± 656	59.8 ± 0.5	36.5 ± 0.4	3.7 ± 0.21
							ES1	150.1 ± 1.5	34892 ± 2330	66.3 ± 0.7	32.4 ± 0.7	1.3 ± 0.13
	B.TX623	70a	197	0.51	1.02	1.02	Meiocyte	129 ± 1.3	11215 ± 440	61.8 ± 0.5	33.3 ± 0.6	5 ± 0.25
							ES1	145.3 ± 0.9	29729 ± 2054	64.7 ± 0.6	34 ± 0.6	1.3 ± 0.13
		70d	297	3.70	3.70	3.37	Meiocyte	126.1 ± 2.5	11260 ± 612	61 ± 0.5	33.2 ± 0.7	5.8 ± 0.35
							ES1	146.8 ± 1.7	27776 ± 2135	65.6 ± 1	33 ± 0.9	1.4 ± 0.1
		70e	344	1.45	0.29	1.74	Meiocyte	127.8 ± 1.9	10864 ± 424	61.3 ± 1	32.8 ± 0.8	5.8 ± 0.36
							ES1	145.2 ± 0.9	23196 ± 1339	65.2 ± 0.4	32.4 ± 0.5	2.4 ± 0.21
	TX7000	71a	178	0.00	0.00	1.69	Meiocyte	128.4 ± 2.5	10763 ± 398	63.3 ± 0.5	31.1 ± 0.6	5.5 ± 0.28
							ES1	151.7 ± 0.9	34584 ± 1477	69.3 ± 0.8	29.3 ± 0.8	1.4 ± 0.06
		71b	308	1.30	1.62	1.95	Meiocyte	131.5 ± 2.3	11687 ± 650	62.6 ± 0.6	33 ± 0.8	4.4 ± 0.36
							ES1	150.9 ± 1.3	28417 ± 1726	66.7 ± 0.8	31.8 ± 0.8	1.5 ± 0.08
hybrid	1111	7.1a	251	0.40	0.00	4.78	Meiocyte	122.1 ± 2.2	9785 ± 484	59.3 ± 0.8	34.8 ± 0.9	5.9 ± 0.27
							ES1	145.9 ± 1.6	24180 ± 977	63.3 ± 0.4	35 ± 0.4	1.7 ± 0.14
		7.1b	176	2.84	1.70	8.52	Meiocyte	126.8 ± 1.4	11839 ± 386	61.1 ± 1.3	33.8 ± 1.3	5.1 ± 0.38
							ES1	143 ± 2.3	22289 ± 1794	67 ± 1.1	31.7 ± 1.1	1.4 ± 0.08
		7.1e	244	0.00	0.41	6.15	Meiocyte	123.8 ± 1.1	11026 ± 308	59.2 ± 0.7	35.4 ± 0.5	5.4 ± 0.4

							ES1	148.6 ± 0.9	35358 ± 1375	61.1 ± 0.4	37.4 ± 0.4	1.5 ± 0.14
		7.1o	245	1.63	0.00	4.49	Meiocyte	127.8 ± 1.9	9260 ± 389	61.1 ± 0.7	33.9 ± 0.7	5 ± 0.23
							ES1	146.9 ± 1.2	25833 ± 658	62.9 ± 0.6	35 ± 0.6	2.1 ± 0.17
	IS 12693	8.1a	285	0.70	0.00	4.21	Meiocyte	128 ± 3.2	10884 ± 956	53.8 ± 5.6	41 ± 5.4	5.2 ± 0.42
							ES1	145.9 ± 1.1	23107 ± 1009	66.8 ± 0.5	31.6 ± 0.5	1.6 ± 0.12
		8.1b	182	0.55	2.20	13.74	Meiocyte	129.3 ± 2.4	12335 ± 1052	57.9 ± 0.5	37.1 ± 0.4	5 ± 0.43
							ES1	149.6 ± 1.3	27370 ± 2400	65.9 ± 1	32.8 ± 0.9	1.3 ± 0.12
		8.1c	129	0.00	0.00	0.78	Meiocyte	130.7 ± 1.4	11396 ± 271	59.3 ± 0.6	36.5 ± 0.6	4.2 ± 0.15
							ES1	148.1 ± 1.8	24339 ± 1432	66.3 ± 0.9	31.6 ± 0.9	2.1 ± 0.15
Other subspecies												
verticilliflorum	Adar	75a	68	0.00	0.00	0.00	Meiocyte	123.2 ± 1.2	10060 ± 315	57.3 ± 0.3	37.9 ± 0.4	4.8 ± 0.35
							ES1	145.8 ± 1.7	28632 ± 1416	62.6 ± 0.5	35.9 ± 0.5	1.6 ± 0.18
	C120	7.2a	157	0.00	0.00	3.18	Meiocyte	143.6 ± 1.6	13684 ± 802	55.8 ± 0.7	40 ± 0.6	4.2 ± 0.41
							ES1	151 ± 1.2	24594 ± 1457	61.9 ± 0.8	36.5 ± 0.8	1.7 ± 0.17
		7.2b	210	0.00	0.00	1.90	Meiocyte	144.3 ± 1.2	13983 ± 633	54 ± 0.6	41.9 ± 0.6	4.1 ± 0.25
							ES1	154.8 ± 1	26439 ± 1514	61.5 ± 0.7	37.3 ± 0.7	1.2 ± 0.1
		7.2c	129	0.00	0.00	2.33	Meiocyte	142.1 ± 3	12889 ± 1032	55.3 ± 0.8	39.8 ± 1	4.9 ± 0.46
							ES1	152.5 ± 1.2	24205 ± 1560	61.8 ± 0.7	36.1 ± 0.8	2.1 ± 0.23
	IS 11010	7.5c	231	0.43	0.43	3.46	Meiocyte	128.5 ± 1.9	13205 ± 642	59.1 ± 0.7	36.9 ± 0.6	4 ± 0.11
							ES1	150.5 ± 1.8	33528 ± 1541	65.2 ± 0.6	33.5 ± 0.6	1.3 ± 0.13
	MN3360	72c	469	1.92	0.64	2.13	Meiocyte	139.7 ± 2.3	14958 ± 1002	55.5 ± 0.5	40.9 ± 0.6	3.6 ± 0.32
							ES1	152.9 ± 1.3	30421 ± 1934	64.9 ± 0.6	33.5 ± 0.7	1.7 ± 0.08
		72d	278	0.36	0.00	3.24	Meiocyte	135.7 ± 1.3	11538 ± 525	56.6 ± 0.2	39.2 ± 0.3	4.3 ± 0.15
							ES1	149.3 ± 1	28616 ± 1585	62.4 ± 0.8	35.8 ± 0.8	1.8 ± 0.13
		72e	193	3.63	0.00	1.55	Meiocyte	130.1 ± 1.7	11109 ± 617	56.2 ± 1.3	39.6 ± 1.3	4.2 ± 0.18
							ES1	151.8 ± 1	31732 ± 1872	63.9 ± 0.8	34.8 ± 0.8	1.3 ± 0.06
	IS 3121	73a	250	0.00	1.20	3.60	Meiocyte	120.3 ± 1.8	10230 ± 672	59.3 ± 0.4	35.1 ± 0.5	5.7 ± 0.31
							ES1	140.4 ± 1.3	22349 ± 1152	65.8 ± 0.4	32.4 ± 0.5	1.8 ± 0.12

	73b	328	1.22	2.74	3.05	Meiocyte	126.6 ± 2.1	9815 ± 428	54.3 ± 0.4	40.3 ± 0.3	5.4 ± 0.21
						ES1	143.8 ± 2.3	23675 ± 2072	60.2 ± 1	37 ± 0.8	2.2 ± 0.19
	73c	215	0.47	7.91	3.26	Meiocyte	124.8 ± 2	9254 ± 327	58.2 ± 0.5	35.2 ± 0.6	6.6 ± 0.44
						ES1	137.3 ± 1.6	20838 ± 1975	64.8 ± 0.8	33.3 ± 0.7	1.9 ± 0.17
017A	74a	100	0.00	0.00	1.00	Meiocyte	122.4 ± 1.1	9174 ± 492	56.3 ± 0.3	37.6 ± 0.4	6.1 ± 0.38
						ES1	139.2 ± 2.3	20780 ± 1513	56.9 ± 0.6	40.9 ± 0.6	2.2 ± 0.21
	74b	188	0.00	0.00	0.53	Meiocyte	122.9 ± 1.2	10417 ± 329	58.7 ± 0.3	35.9 ± 0.3	5.5 ± 0.31
						ES1	137.3 ± 0.9	20689 ± 797	63 ± 0.7	35.1 ± 0.8	2 ± 0.14
IS 12702	76a	191	18.85	1.57	15.71	Meiocyte	130.8 ± 2.6	13920 ± 973	52.3 ± 1.1	42.4 ± 1.1	5.2 ± 0.42
						ES1	149.6 ± 2	29975 ± 1234	63.1 ± 0.8	35.4 ± 0.8	1.6 ± 0.16
	76b	165	4.24	0.00	10.30	Meiocyte	132 ± 1.6	14523 ± 656	51.2 ± 0.6	42.9 ± 0.5	5.9 ± 0.39
						ES1	142.7 ± 1.6	40873 ± 2351	61.1 ± 0.7	37.3 ± 0.8	1.3 ± 0.15
	76d	267	44.94	13.86	20.97	Meiocyte	125.6 ± 2.2	13382 ± 458	56.4 ± 0.7	38.3 ± 0.6	5.2 ± 0.41
						ES1	144.9 ± 1.2	39833 ± 2423	68.8 ± 0.8	29.5 ± 0.7	1.7 ± 0.13
IS 12699	77b	284	0.00	0.00	0.70	Meiocyte	139.5 ± 1	8410 ± 390	45.5 ± 0.6	49.9 ± 0.6	4.6 ± 0.54
						ES1	153.8 ± 1	28954 ± 1219	62.1 ± 0.8	36.3 ± 0.8	1.6 ± 0.14
	77c	630	0.32	0.16	0.63	Meiocyte	135.9 ± 1.9	10620 ± 497	46.8 ± 0.7	48.9 ± 0.8	4.4 ± 0.31
						ES1	150.6 ± 1.2	30491 ± 1382	64.7 ± 0.7	34 ± 0.7	1.3 ± 0.05
R-319	8.2b	146	0.00	0.00	0.68	Meiocyte	119.7 ± 2	17263 ± 1076	50.3 ± 3.7	46.2 ± 3.7	3.5 ± 0.2
						ES1	144.1 ± 1.1	35495 ± 2194	60 ± 0.5	38.8 ± 0.5	1.2 ± 0.06
	8.2c	189	0.00	0.00	0.53	Meiocyte	130.2 ± 1.7	12012 ± 579	58.7 ± 0.6	37.4 ± 0.7	4 ± 0.17
						ES1	140.9 ± 1	29463 ± 1109	65.5 ± 0.8	32.9 ± 0.7	1.6 ± 0.13
26	81b	159	0.00	0.63	0.63	Meiocyte	136.1 ± 2	12148 ± 538	57.1 ± 0.6	38.9 ± 0.5	4 ± 0.28
						ES1	150.9 ± 1	34757 ± 2196	64.9 ± 0.6	34 ± 0.6	1.1 ± 0.15
	81c	477	1.05	0.42	2.31	Meiocyte	140.4 ± 1.4	18119 ± 1253	60.9 ± 0.5	36.1 ± 0.5	3 ± 0.24
						ES1	151.3 ± 0.6	34010 ± 2494	68.1 ± 0.6	31 ± 0.6	0.9 ± 0.11
Yabia	82a	108	0.00	0.00	0.93	Meiocyte	128.1 ± 1.6	12887 ± 779	56.4 ± 0.7	39.5 ± 0.8	4.1 ± 0.29
						ES1	143.3 ± 1.7	26418 ± 1594	63.2 ± 0.8	35.3 ± 0.8	1.5 ± 0.13
PI 369487	83a	367	2.18	3.00	10.90	Meiocyte	131.1 ± 1.9	8371 ± 535	60.1 ± 0.4	33.4 ± 0.7	6.5 ± 0.43

						ES1	148.3 ± 2.1	30604 ± 3599	65.3 ± 0.9	32.3 ± 0.9	1.6 ± 0.2
	83b	324	2.47	2.16	15.12	Meiocyte	134.2 ± 2.1	13635 ± 1002	63 ± 0.6	32.7 ± 0.5	4.2 ± 0.26
						ES1	150.3 ± 1.3	32120 ± 2235	65.2 ± 1	33.2 ± 0.9	1.6 ± 0.21
	83c	404	3.22	1.98	14.36	Meiocyte	134.7 ± 2.4	15841 ± 1930	61.9 ± 0.4	34.1 ± 0.5	4 ± 0.37
						ES1	150 ± 1.2	33435 ± 1357	68.3 ± 0.6	30.5 ± 0.6	1.1 ± 0.14
IS 12472	96a	279	2.51	0.36	7.53	Meiocyte	134.2 ± 2.1	12059 ± 993	57.9 ± 1.1	36.8 ± 1.1	5.3 ± 0.31
						ES1	150.5 ± 1.5	30880 ± 1298	60.6 ± 0.7	37.6 ± 0.8	1.8 ± 0.11
