# Supplementary material for: Apospory appears to accelerate onset of meiosis and sexual embryo sac formation in sorghum ovules
Source: BMC Plant Biol. 2011 Jan 11;11:9. doi: 10.1186/1471-2229-11-9 (PMC3023736; doi:10.1186/1471-2229-11-9)
Supplement: Additional file 4 — Abbreviated ANOVA table for data summarized in Figure 2. Two analyses were performed, one for all 116 S. bicolor genotypes listed in Additional file 3 and one that included only genotypes from accessions of Additional file 3 represented by two or more genotypes. [file 1471-2229-11-9-S4.PDF]

Additional file 4

Source (by model)	df	*F*-value[a] Ovule curvature	Ovule area	Nucellus percent	Integument percent	Germ cell percent
All 116 plants						
Taxonomic group (T)	6	58.5***	14.2***	181.4***	167.1***	7.1***
Stage (S)	1	4631.9***	6574.9***	2265.4***	459.3***	7781.3***
T x S	6	6.4***	8.3***	13.8***	14.8***	0.5 (NS)
Genotypes within T	109	15.3***	7.8***	31.4***	29.4***	7.1***
Limited to multiple plants per accession						
T	6	62.6***	17.7***	229.6***	214.8***	11.7***
S	1	3677.8***	5234.6***	1787.6***	363.3***	6181.3***
T x S	6	9.1***	8.7***	16.2***	17.4***	1.6 NS
Accessions (A) within T	28	39.3***	15.0***	63.6***	61.9***	12.7***
Genotypes within A	59	4.6***	3.8***	8.7***	8.1***	3.5***

[a] ***, $P < 0.001$
