# Supplementary material for: Apospory appears to accelerate onset of meiosis and sexual embryo sac formation in sorghum ovules
Source: BMC Plant Biol. 2011 Jan 11;11:9. doi: 10.1186/1471-2229-11-9 (PMC3023736; doi:10.1186/1471-2229-11-9)
Supplement: Additional file 5 — Frequency of aposporous initials (AI), aposporous embryo sacs (AES) and large stack cells (LSC) in ovules of 34 S. bicolor genotypes from 27 accessions (see Additional file 9 for accession information). Genotypes listed here supplement those listed in Additional file 3 for the AI, AES and LSC tally. [file 1471-2229-11-9-S5.PDF]

Additional file 5

Taxonomic description	Accession common name	Genotype	n	Frequency (%)		
				AI	AES	LSC
Race (subspecies bicolor)						
bicolor	4528	79c	82	0.00	0.00	0.00
caudatum	015A	98a	82	0.00	0.00	0.00
		98c	96	0.00	0.00	1.04
	Ba Ye Qi	13.1e	90	4.44	0.00	15.56
	Gadyabo	97a	63	0.00	0.00	0.00
durra	Chaondera	11.1b	171	4.09	0.58	0.00
		11.1c	76	0.00	0.00	0.00
	Mashila	100b	192	0.00	0.00	0.52
		100c	123	0.00	0.00	1.63
	PI 330838	101b	96	0.00	0.00	3.13
	Vir-5049	9.1b	119	0.00	0.00	0.00
durra-caudatum	Westland	5.2b	217	0.92	0.00	1.84
guinea	Sokombe	89a	209	0.00	0.00	0.00
	Tchari	91b	184	1.09	0.54	2.17
		91c	61	1.64	0.00	1.64
kafir	IS 28865	92c	74	0.00	0.00	8.11
	IS 2942	2.1a	197	2.54	0.00	5.58
		2.1b	107	0.93	0.93	3.74
	Lydenburg Red	10.1d	51	5.88	1.96	7.84
breeding line	B.TX642	34c	98	0.00	0.00	1.02
	Piper	36a	55	0.00	0.00	9.09
	TX2536	48a	179	2.23	0.56	1.68
	TX378	21a	107	0.00	0.00	0.00
	TX7078	26a	147	0.00	0.00	0.68
		26b	147	0.68	0.00	0.00
		26c	71	0.00	0.00	0.00
Other subspecies						
verticilliflorum	Adar	75c	121	0.00	0.83	0.00
	26	81a	62	22.58	0.00	11.29
	017A	74c	240	0.42	1.25	5.83

IS 11010	7.5a	80	0.00	0.00	2.50
IS 12699	77a	94	0.00	3.19	1.06
IS 12702	76c	265	14.72	4.91	10.19
PI 369493	78a	106	0.00	1.89	13.21
R-319	8.2a	155	0.00	0.00	0.00
