# Supplementary material for: Apospory appears to accelerate onset of meiosis and sexual embryo sac formation in sorghum ovules
Source: BMC Plant Biol. 2011 Jan 11;11:9. doi: 10.1186/1471-2229-11-9 (PMC3023736; doi:10.1186/1471-2229-11-9)
Supplement: Additional file 6 — Abbreviated ANOVA table for morphometric comparisons among accessions that were clustered based on frequency aposporous embryo sac (AES) formation. The data are summarized in Figure 5A. [file 1471-2229-11-9-S6.PDF]

Additional file 6

		F-value[a]				
**Source**	**df**	**Ovule curvature**	**Ovule area**	**Nucellus percent**	**Integument percent**	**Germ cell percent**
AES cluster (AES-C)	3	47.366***	25.834***	50.328***	39.083***	16.967***
Stage (S)	1	1305.680***	1598.450***	674.796***	98.275***	3024.533***
AES-C x S	3	1.422 NS	8.262***	0.813 NS	2.649*	10.528***
Genotype within AES-C	111	15.633***	7.089***	38.847***	36.304***	6.809***

[a] *, $P < 0.05$; ***, $P < 0.001$; NS, not significant
