# Supplementary material for: Apospory appears to accelerate onset of meiosis and sexual embryo sac formation in sorghum ovules
Source: BMC Plant Biol. 2011 Jan 11;11:9. doi: 10.1186/1471-2229-11-9 (PMC3023736; doi:10.1186/1471-2229-11-9)
Supplement: Additional file 7 — Abbreviated ANOVA table for ovule curvature comparisons among F2 that were clustered based on frequency aposporous embryo sac (AES) formation. The data are summarized in Figure 5B. Also listed are ANOVA F-ratios for mean AES frequency comparisons made between groups of F2 genotypes clustered by ovule curvature (angle) at the meiocyte (dyad through early tetrad), 1-nucleate embryo sac (ES1), and 8-nucleate embryo sac (ES8) stages. [file 1471-2229-11-9-S7.PDF]

Additional file 7

Dependent variable	r^2	Source	df	*F*-ratio[a]
Ovule curvature	0.82	AES Cluster (AES-C)	3	11.06***
		Stage (S)	2	5955***
		AES-C x S	6	3.944***
		Genotype within AES-C	296	11.31***
AES	0.007	Meiocyte angle cluster	3	0.648
AES	0.001	ES1 angle cluster	3	0.085
AES	0.004	ES8 angle cluster	3	0.441

[a] ***, $P < 0.001$
