# Supplementary material for: Apospory appears to accelerate onset of meiosis and sexual embryo sac formation in sorghum ovules
Source: BMC Plant Biol. 2011 Jan 11;11:9. doi: 10.1186/1471-2229-11-9 (PMC3023736; doi:10.1186/1471-2229-11-9)
Supplement: Additional file 8 — Abbreviated ANOVA table for ovule curvature comparisons among RIL that were clustered based on frequency aposporous initial (AI) or aposporous embryo sac (AES) formation. The data are summarized in Figure 5C (top and bottom graphs). Also listed are ANOVA F-ratios for mean AI or AES frequency comparisons made between groups of RIL clustered by ovule curvature (angle) at the meiocyte (dyad through early tetrad) and 1-nucleate embryo sac (ES1) stages. [file 1471-2229-11-9-S8.PDF]

Additional file 8

Dependent variable	r^2	Source	df	*F*-ratio[a]
Ovule curvature	0.70	AES cluster (AES-C)	2	148.8***
		S	1	2377.7***
		AES-C x S	2	6.821**
		Genotype within AES- C	116	26.9***
	0.69	AI cluster (AI-C)	2	66.2***
		Stage (S)	1	3143.4***
		AI-C x S	2	0.982
		Genotype within AI-C	116	28.2***
AI	0.05	Meiocyte angle cluster	2	3.440*
	0.03	ES1 angle cluster	2	2.476
AES	0.04	Meiocyte angle cluster	2	2.654
	0.11	ES1 angle cluster	2	7.325**

[a] *, **, ***, $P < 0.05$, $P < 0.01$, $P < 0.001$
