# Supplementary material for: Apospory appears to accelerate onset of meiosis and sexual embryo sac formation in sorghum ovules
Source: BMC Plant Biol. 2011 Jan 11;11:9. doi: 10.1186/1471-2229-11-9 (PMC3023736; doi:10.1186/1471-2229-11-9)
Supplement: Additional file 9 — Race or subspecies, common name, collection identifiers and country of origin for 72 Sorghum bicolor accessions evaluated for apomictic embryo sac formation and/or other morphometric variables of ovule development. [file 1471-2229-11-9-S9.PDF]

Additional file 9

Taxonomic description	Common name	USDA no.	ICRISAT no.	Origin
Race (subsp. *bicolor*)				
bicolor	410	PI 520795		Kenya
bicolor	4528	PI 543172		Yemen
bicolor	Farfara	PI 537086		Niger
bicolor	Mukaruki	PI 482838		Zimbabwa
caudatum	015A	PI 520775		Kenya
caudatum	Agira	PI 217855	IS 2324	Sudan
caudatum	Ba Ye Qi		IS 37024	China
caudatum	Gadyabo	PI 514464		Senegal
caudatum	IS 11888	PI 330168	IS 11888	Ethiopia
caudatum	Nian Gaoliang		IS 37043	China
durra	Aispuri	PI 253638		India
durra	Aispuri-C	PI 533817	IS 1151C	India
durra	Chaondera	PI 570864	IS 4018	Sudan
durra	Colby	PI 571105	IS 9683	Sudan
durra	Gharib		IS 29053	Yemen
durra	IS 13856	PI 308295	IS 13856	South Africa
durra	IS 36854		IS 36854	Yemen
durra	Karad Local	PI 248318		India
durra	Karad Local-C	PI 533932	IS 1122(A)C	India
durra	Mashila	PI 329425	IS 11182	Ethiopia
durra	PI 330838	PI 330838	IS 12122	Ethiopia
durra	Vir-5049	PI 562347		Sudan
durra-caudatum	Early Kalo	NSL 3999	IS 851	
durra-caudatum	Westland	NSL 4003	IS 836	

guinea	Baba Founfoun	PI 513915		Benin
guinea	Bassi Wende	PI 514349		Senegal
guinea	Keninke Teli	PI 525511		Mali
guinea	Sokombe	PI 513771		Niger
guinea	Tchari	PI 515583		Togo
kafir	Combine Kafir	PI 571057	IS 9627	Sudan
kafir	Dewe	PI 482747		Zembabwa
kafir	IS 28865	PI 475573	IS 28865	Yemen
kafir	IS 2942	PI 643564	IS 2942	
kafir	IS 3922	PI 644295	IS 3922	
kafir	Lydenburg Red	PI 229862	IS 2386	South Africa
kafir	White Kafir	PI 48770	IS 197	South Africa
Breeding line	1111	PI 542649		Algeria
Breeding line	ARG1			
Breeding line	B.Tx623	PI 564163		
Breeding line	B.Tx642			
Breeding line	Combine Shallu	PI 562711	IS 475	
Breeding line	IS 12693	PI 225905	IS 12693	Zambia
Breeding line	IS 3620C	PI 533839	IS 3620C	Nigeria
Breeding line	O756	PI 302166		Australia
Breeding line	Piper			
Breeding line	QL36			
Breeding line	TX2536			
Breeding line	TX378 (Redian)			
Breeding line	TX2536			
Breeding line	TX2737	CSR 234		
Breeding line	TX2741			
Breeding line	TX3042			

Breeding line	TX378			
Breeding line	TX414			
Breeding line	TX7000			
Breeding line	TX7078			
Breeding line	Zhuronskiya	PI 539065		Kazakhstan
Other subspecies				
verticilliflorum	Adar	PI 524512		Sudan
verticilliflorum	24	PI 369493		Nigeria
verticilliflorum	26	PI 369484		Ghana
verticilliflorum	017A	PI 520777		Kenya
verticilliflorum	C120	PI 535995		Cameroon
verticilliflorum	IS 11010	PI 329252	IS 11010	Ethiopia
verticilliflorum	IS 12472	PI 563513	IS 12472	Sudan
verticilliflorum	IS 12699	PI 302118	IS 12699	Ethiopia
verticilliflorum	IS 12702	PI 302267	IS 12702	Tanzania
verticilliflorum	IS 3121	PI 226096	IS 3121	Kenya
verticilliflorum	MN3360	PI 208190		South Africa
verticilliflorum	PI 369487	PI 369487		Nigeria
verticilliflorum	PI 369493	PI 369493		Nigeria
verticilliflorum	R-319	PI 329251		Ethiopia
verticilliflorum	Yabia	PI 524718		Sudan
